# Sensory neuron dysfunction in orthotopic mouse models of colon cancer

**DOI:** 10.1186/s12974-022-02566-z

**Published:** 2022-08-12

**Authors:** Mihály Balogh, Jixiang Zhang, Caitlyn M. Gaffney, Neha Kalakuntla, Nicholas T. Nguyen, Ronnie T. Trinh, Clarissa Aguilar, Hoang Vu Pham, Bojana Milutinovic, James M. Nichols, Rajasekaran Mahalingam, Andrew J. Shepherd

**Affiliations:** 1 The MD Anderson Pain Research Consortium and the Laboratories of Neuroimmunology, Department of Symptom Research, Division of Internal Medicine, The University of Texas MD Anderson Cancer Center, Houston, TX USA; 2 Pharmaceutical Analysis, Groningen Research Institute of Pharmacy, University of Groningen, 9700 AD Groningen, The Netherlands; 3 Graduate School of Biomedical Sciences, UT Southwestern Medical Center, Dallas, TX USA; 4 Neuroscience and Behavior Graduate Program, University of Massachusetts Amherst, Amherst, MA USA; 5 Department of Neurosurgery, Division of Internal Medicine, The University of Texas MD Anderson Cancer Center, Houston, TX USA

**Keywords:** Colon cancer, Neuropathy, Paraneoplastic neuropathy, DRG neuron, Neuropathic pain

## Abstract

Reports of neurological sequelae related to colon cancer are largely restricted to rare instances of paraneoplastic syndromes, due to autoimmune reactions. Systemic inflammation associated with tumor development influences sensory neuron function in other disease models, though the extent to which this occurs in colorectal cancer is unknown. We induced orthotopic colorectal cancer via orthotopic injection of two colorectal cancer cell lines (MC38 and CT26) in two different mouse strains (C57BL/6 and Balb/c, respectively). Behavioral tests of pain sensitivity and activity did not detect significant alterations in sensory sensitivity or diminished well-being throughout tumor development. However, immunohistochemistry revealed widespread reductions in intraepidermal nerve fiber density in the skin of tumor-bearing mice. Though loss of nerve fiber density was not associated with increased expression of cell injury markers in dorsal root ganglia, lumbar dorsal root ganglia neurons of tumor-bearing animals showed deficits in mitochondrial function. These neurons also had reduced cytosolic calcium levels in live-cell imaging and reduced spontaneous activity in multi-electrode array analysis. Bulk RNA sequencing of DRGs from tumor-bearing mice detected activation of gene expression pathways associated with elevated cytokine and chemokine signaling, including CXCL10. This is consistent with the detection of CXCL10 (and numerous other cytokines, chemokines and growth factors) in MC38 and CT26 cell-conditioned media, and the serum of tumor-bearing mice. Our study demonstrates in a pre-clinical setting that colon cancer is associated with latent sensory neuron dysfunction and implicates cytokine/chemokine signaling in this process. These findings may have implications for determining risk factors and treatment responsiveness related to neuropathy in colorectal cancer.

**Supplementary Information:**

The online version contains supplementary material available at 10.1186/s12974-022-02566-z.

## Introduction

Colorectal cancer, being the third-most common form of cancer, accounts for approximately 10% of cancer-related mortality [1]. In 2013, 771,000 people died of colorectal cancer globally [2]. Although colon cancer-related mortality and incidence has been decreasing slowly but steadily, hundreds of thousands of people in the USA alone are still affected yearly [3]. Therefore, new non-cytotoxic therapeutics are continuously developed (such as checkpoint inhibitors and other immunological modulators), but cytotoxic agents such as oxaliplatin are still a cornerstone of colon cancer treatment regimens [1, 4]. Current use of oxaliplatin-based regimens often comes with dose-limiting side effects and prolonged, debilitating neuropathy [4].

Most cancers are capable of producing cytokines, growth factors, chemotactic molecules, and proteases, hence influencing or inducing systemic inflammation and hypercoagulability [5, 6]. In addition to their roles in tumor growth, some of these mediators may also play important roles in the development of neuropathic pain, e.g., complement 5 (C5), or the CXCR3 receptor, and its ligand CXCL10 [7, 8]. These modulators have been shown to be upregulated in experimental animals or patients suffering from colon cancer [9–13]. Taking these and other observations together, there is now clear evidence showing shared systemic processes involved in both the development of cancer and neuropathic pain [14].

Traditionally, paraneoplastic neuropathy (where clinical presentation of neuropathy symptoms is induced directly by cancer) is regarded as a rare complication [15, 16], and is typically characterized as a form of autoimmune or chronic inflammatory demyelinating polyneuropathy. What has not been explored as extensively is how widespread earlier symptoms of neuronal dysfunction may be. This is important because pre-existing neuropathy is a risk factor for subsequent development of neuropathic pain [17].

Despite these observations, the majority of pre-clinical data on neuropathic pain, including chemotherapy induced peripheral neuropathy (CIPN), are gained from studies where otherwise naïve animals are dosed with chemotherapy. Few studies have investigated neuronal dysfunction directly induced by tumor growth. Therefore, we set out to determine the extent to which preclinical models of colorectal cancer affect sensory neuron function, and by what means. We used two distinct mouse orthotopic colon cancer models: MC38 cells in C57BL/6 mice, and CT26 cells in Balb/c mice. After confirming engraftment, we investigated overall indicators of animal well-being (weight change, nestlet shredding assay), the development of typical neuropathic and tumor-related pain symptoms (mechanical sensitivity, cold allodynia, abdominal pain), and performed immunohistochemistry to investigate changes in intraepidermal nerve fiber (IENF) density, as one of the prominent symptoms of neuropathy. Neuronal dysfunction was also analyzed with the use of a mitochondrial function assay and immunohistochemistry on dorsal root ganglia from tumor-bearing and control animals. To further analyze neuronal function, intracellular Ca^2+^ levels and electrical activity of DRG neurons were compared by live-cell Ca^2+^ imaging and multi-electrode array analysis, respectively. Finally, utilizing cytokine protein arrays, and bulk RNA sequencing of dorsal root ganglia, we aimed to characterize tumor-induced changes in circulating factors and draw connections to transcriptional changes at the level of the DRG. Collectively, our data suggest that neuronal dysfunction could represent a latent but common complication of colorectal cancer, which could represent a potential risk factor for subsequent development of neuropathy, induced by chemotherapy or otherwise.

## Methods

### Generation and maintenance of luciferase-expressing MC38 and CT26 CRC cells

The MC38 colon adenocarcinoma cell line (C57BL/6 origin) was obtained from Kerafast, Boston, USA (catalog # ENH204-FP). Cells were maintained in 10 cm TC dishes at 37 °C, 5% CO_2_. Cell culture media: Dulbecco's modified MEM (DMEM) with 10% fetal bovine serum (FBS), 2 mM glutamine, 0.1 mM nonessential amino acids, 1 mM sodium pyruvate, 10 mM HEPES, 1 × penicillin/streptomycin. Firefly luciferase-expressing CT26 colon carcinoma cells (Balb/c origin) were obtained from Cellomics (catalog #: SC-1298) and maintained at 37 °C and 5% CO_2_ in RPMI-1640 Medium with 10% FBS and 1 × penicillin/streptomycin.

In order to non-invasively measure tumor growth in C57BL/6 mice, we transduced MC38 cells with a lentivirus (catalog # FCT160; Kerafast, Boston, USA) containing the firefly luciferase (CMV) and neomycin resistance genes. Our procedure was based on the provider’s protocol [18]. Briefly, cells were plated in six well cell culture plate in 2 ml growth medium. After cell growth reached ~ 80% confluence (48 h), growth medium was aspirated and 2 ml transduction medium was added, containing 7.5 µl (approx. 7.5 × 10^5^ CFU) of the virus. 24 h later, cells were selected for infection by adding 400 µg/ml G418 to the growth medium [19]. This concentration was the minimum required to kill all uninfected MC38 control wells within 7 days. All clones were expanded and stocks cryopreserved. Firefly luciferase expression was verified in vitro with the addition of 150 µg/ml luciferin (catalog #: LUCK-1G; Goldbio) immediately prior to reading luminescence with a Biotek Synergy plate reader.

### Animals

All experiments and procedures involving the use of mice were approved by the University of Texas MD Anderson Cancer Center Animal Care and Use Committee at MD Anderson Cancer Center (Houston, TX, USA) in accordance with the National Institutes of Health’s *Guide for the Care and Use of Laboratory Animals*. Every effort was made to minimize the number of mice used and their suffering. Animals were randomly assigned to individual experimental groups. 8- to 14-week-old male mice (weighing 22–30 g) were used for each experiment. Male C57BL/6J (catalog #: 000664) and BALB/c (catalog #: 000651) mice were obtained from Jackson Laboratories and kept under 12:12 light:dark cycle (07:00 to 19:00 h) with access to food and water ad libitum*.* All mice were habituated to the housing environment for at least 4 days before experiments began.

### Orthotopic colon cancer models: local MC38 and CT26 cell injections

Colon tumors were induced using minimally invasive transanal rectal injection, based on the methods of Donigan et al [20]. Under isoflurane anesthesia (3% isoflurane in oxygen), 2 × 10^5^ luciferase-expressing MC38 or 1 × 10^5^ luciferase-expressing CT26 cells in 50 µl growth medium were injected using a 0.3 ml (U30) insulin syringe, into the colorectal wall of C57BL/6 and BALB/c mice, respectively. Control mice were anesthetized and injected with 50 µl vehicle (cell growth medium) in the same manner. No animals died or had to be euthanized as a result of tumor development during the 4-week experimental period.

### IVIS Lumina in vivo bioluminescent imaging: non-invasive tumor growth measurements

Tumor growth was followed by in vivo non-invasive bioluminescent imaging (BLI) using an IVIS XR Lumina (PerkinElmer), as described previously, with slight modifications [21]. Mice were injected with D-luciferin (150 mg/kg, i.p. dissolved in sterile PBS). After 10 min, a 1-min exposure of the ventral aspect was captured. Luminescence was quantified within a circular ROI overlying the colorectal region for each animal and expressed as average radiance (p/s/cm^2^/sr) by the Living Image software (PerkinElmer). Tumor- and vehicle-injected mice were measured weekly. Animals not developing tumors (as indicated by elevated signal intensity of at least 500 p/s/cm^2^/sr on the second week after injection) were excluded from experiments and did not exhibit visible tumor development upon necropsy (less than 5% of mice, data not shown).

### Behavioral assays

All behavioral assays were performed in the same room as which mice were housed. Before the start of any experiment, mice were habituated to the testing apparatus for 2 days prior to initiating testing. Experimenters were blinded to tumor/vehicle injection to the fullest extent possible, however, 4 weeks post-injection, swelling of the tumor mass in the perianal area was often readily apparent. All behavioral testing was carried out during the light cycle, between 08:00 and 12:00.

#### Nestlet shredding test

In order to assess the extent to which pain sensitivity could be obfuscated by debilitating effects of late-stage tumor growth, we employed a nestlet shredding assay [22], a well-described indicator of well-being and a physiological behavior in which healthy mice are motivated to engage [23, 24]. 24 h before testing began, nesting material was removed from the home cages. The following day, mice were placed in individual cages and habituated for 1 h. After habituation, weighed nestlets (2-inch square) were place into the cages. The percentage of the nestlet that was shredded and/or became detached from the original nestlet was calculated 3 h later.

#### Abdominal sensitivity

An abdominal von Frey filament assay was performed to assess abdominal sensitivity changes induced by tumor growth [25]. Mice were placed in single-occupancy Plexiglas boxes, situated on a wire mesh platform. After 30 min of habituation, withdrawal behaviors were counted (withdrawal or licking of the abdomen as a result of stimulation, or whole-body withdrawal) in response to application of a 0.02 g von Frey filament to the lower abdomen. Each mouse was measured 5 times, with intervals of ~ 10 s. After a 1-min rest period this was repeated, resulting in a total of 10 filament applications per mouse. For each application, responses were scored as: 0 = no response; 1 = immediate slight attempt to escape or light licking or scratching of the stimulated site; 2 = intense withdrawal of the abdomen or jumping. The cumulative scores of the ten measurements are presented for each animal.

#### Behavioral test of sensorimotor function

The adhesive tape removal test of sensorimotor acuity was conducted as described previously [26]. Briefly, a 5 mm × 5 mm piece of electrical tape was applied to the center of the plantar surface of each hindpaw immediately before placement in single-occupancy Plexiglas boxes for observation. The time to initial contact with either piece of tape and the latency to removal of the tape from the hindpaw were recorded by blinded observers. Mice were habituated to restraint and to the test conditions 10 and 12 days post-tumor injection, before undergoing testing every 2 days for the next 10 days.

#### Hindpaw mechanical and cold sensitivity

Before each experiment, animals were acclimated to the environment for at least 30 min on every testing day, and the day before the first testing. For the assessment of hindpaw mechanical allodynia, mice were placed in single-occupancy Plexiglas boxes, situated on a wire mesh platform. Von Frey filaments of increasing strength (0.04–2 g) were applied to the plantar surface of each hind paw until the filament was bent, starting with the 0.6 g filament in an up–down paradigm [27]. 50% paw withdrawal threshold values were calculated for each animal [27–29]. Measurements were performed before, and weekly after tumor cell injections for 4 weeks. Values are presented as the average percentage change compared to baseline. Cold sensitivity of mice was measured using powdered dry ice, based on [30]. Mice were habituated to single-occupancy Plexiglas boxes situated on a 6.35-mm-thick pane of glass. Powdered dry ice compacted into a 3-ml syringe was applied to the glass directly underlying the plantar surface of each hind paw. Withdrawal latencies to this cooling stimulus were recorded in seconds using a stopwatch, and each paw was measured two times (with > 3 min between tests) and the mean latency calculated.

### Immunohistochemistry

Dorsal root ganglia and 3-mm plantar punch biopsies of hind paws and fore paws were harvested after perfusion with 4% paraformaldehyde (PFA) in phosphate buffer (PB), pH 7.4, as described previously [31, 32]. At least 3 biological replicates/groups were measured from at least two different cohorts of mice for all tissues.

Plantar tissues were post-fixed in 4% PFA overnight before being transferred into 15% sucrose and 10% EDTA in PBS for 48 h for decalcification of bones, as described previously [33, 34]. After further incubation in 30% sucrose overnight, 40 µm lateral sections (skin to skin) were prepared with a Leica CM3050S cryostat and collected onto microscope slides. Immunohistochemistry was performed as previously described [31, 32]. After three 5-min washes with 0.1 M PB, slides containing sections were blocked and permeabilized with 10% goat serum, and 0.3% Triton X-100 in 0.1 M PB for 1 h at 4 °C. Sections were then incubated overnight at 4 °C in primary antibodies diluted in blocking buffer (rabbit anti-PGP9.5: Thermo Fisher Scientific, catalog #38-1000; dilution: 1:500; rat anti-CD68, Bio-Rad, catalog #MCA1957GA; dilution: 1:300). The following day samples were washed 3 times in blocking buffer and then incubated with secondary antibody (1:1000 IgG-Alexa Fluor 488 goat anti-rabbit and goat anti-rat 568 antibody in blocking buffer and 1 µg/ml DAPI for 4 h, protected from light at 4 °C. Slides were then washed 3 times in 0.1 M PB, mounted with ProLong Gold anti-fade reagent, cover-slipped, sealed with nail polish and kept at − 20 °C until imaging with a Nikon Ti2 confocal microscope. Images were taken at 20 × magnification. Images are a composite of 10 focal planes in a 40-µm z-stack at 4 µm increments. Tissue samples from at least 3 mice/experimental group were imaged; 3–4 different sections were imaged from the same animal. Following image acquisition, intraepidermal nerve fiber (IENF) density was determined in the plantar skin as described previously [35–37] with the Nikon NIS-Elements Imaging Software and presented as number of IENFs/100 µm. CD68 signal intensity of the same tissue samples was determined in the hind paw skin by the application of ROIs from the edge of the plantar skin to 200 µm below the epidermis.

DRG samples were harvested and handled similarly to plantar punch tissue samples, as described previously [21, 31, 33]. Lumbar DRGs were harvested under a dissection microscope into 4% PFA, then placed into PBS containing 15% and 30% sucrose solutions in PBS, each overnight. 25-µm sections were stained using the same procedure as for plantar skin samples. For the stains performed using NF200, 1% BSA was added to the blocking buffer. After blocking for 1 h, sections were washed with PBS-Tween 20 (0.05%) and blocked with Goat F(ab) anti-mouse IgG H&L (Abcam, catalog#ab6668; dilution 1:50) in PBS for 1 h. The sections were rinsed again with PBS-Tween20 before the primary antibody was added in blocking buffer. The following primary antibodies were used for DRG immunofluorescence: rabbit anti-activating transcription factor 3 (ATF-3; Novus Biologicals, catalog #NBP1-85816; dilution: 1:300), rat anti-CD68 (Bio-Rad, catalog #MCA1957GA; dilution: 1:300), rabbit anti-CXCL10 (Thermo Fisher Scientific, catalog #701225; dilution 1:200), rabbit anti-CXCR3 (Thermo Fisher Scientific, catalog #PA5-23104; dilution 1:200), and mouse anti-NF200 (Sigma-Aldrich, catalog #N0142; dilution 1:200). Secondary antibodies: IgG-Alexa Fluor 594 goat anti-rabbit, IgG-Alexa Fluor 488 goat anti-rat (dilution: 1:1000), IgG-Alexa Fluor 488 goat anti-rabbit (dilution: 1:500), and IgG-Alexa Fluor 555 goat anti-mouse (dilution: 1:500). DAPI (1 µg/ml) was added together with the secondary antibodies.

### Mitochondrial function assay

To determine tumor-related effects on neuronal mitochondrial bioenergetics, DRGs of tumor-bearing and control animals were investigated by the XF^e^24 Extracellular Flux Analyzer (Seahorse Bioscience), as described previously [35]. Immediately after collection of lumbar (L2–L5/6) DRG from tumor-bearing and control mice into Ham’s F10 media (Thermo Fisher Scientific, USA), connective tissue was removed, ganglia were enzymatically dissociated, and an enriched neuronal fraction was obtained [38]. Cells were plated into poly-DL-polyornithine (0.01%)/laminin (2 µg/mL)-coated XF24 plates (Seahorse Bioscience, Santa Clara, CA) in Ham’s F10 medium containing N2 supplement without insulin at approximately 1,000 cells/well (Invitrogen). 24 h after plating, oxygen consumption rate (OCR) and the extracellular acidification rate (ECAR) was analyzed by the application of an ATP synthase inhibitor (2 µM oligomycin), a protonophore that dissipates the proton gradient across the inner mitochondrial membrane (4 µM carbonyl cyanide p-trifluoromethoxyphenylhydrazone, FCCP), and the combination of a complex I and II inhibitor (2 µM rotenone and antimycin A, respectively). This enables determination of the portion of basal OCR coupled to ATP synthesis, the maximal respiratory capacity, and the amount of non-mitochondrial respiration [35, 39]. Dissociated DRGs were measured in an assay cycle of [3 min mixing, 2 min pause, 3 min measurement], repeated 3 times. Data were normalized to total protein levels, measured by BCA Protein Assay according to the manufacturer’s instructions (Thermo Scientific; USA).

### Live-cell calcium imaging

The live-cell calcium imaging (Ca^2+^ imaging) experiments were performed on cultured mouse DRG neurons as previously described [21, 40]. DRGs were collected from C57BL/6 mice as detailed in [41]. Isolated DRGs were digested with collagenase and pronase (2 mg/ml and 1 mg/ml in DMEM, respectively). Before plating, the prepared cell suspension was centrifuged through a 1:1 28% and 12.5% Percoll density gradient (3900 rpm for 10 min), in order to remove non-cellular debris [42]. Thereafter, cells were plated onto poly-L-ornithine- and laminin-coated 12 mm circular glass coverslips and incubated in 1:1 TNB media supplemented with protein–lipid complex (Biochrom) and DMEM + 10% FBS at 37 °C in 5% CO_2_ for 1 to 2 days. Calcium imaging was conducted using Fura 2-AM, as described previously [21, 43]. Briefly, DRG neuron coverslips were incubated with the Ca^2+^-sensitive dye Fura-2- AM (Invitrogen, 2 μM) at room temperature, for 20 min, dissolved in HEPES-buffered HBSS (‘HH buffer’) containing the following (in mM): 140 NaCl, 5 KCl, 1.3 CaCl_2_, 0.4 MgSO_4_, 0.5 MgCl_2_, 0.4 KH_2_PO_4_, 0.6 NaHPO_4_, 3NaHCO_3_, 10 glucose, and 10 HEPES adjusted to pH 7.4 with NaOH and adjusted to 310 mOsm with sucrose. Coverslips were then placed in an MS-512SP recording chamber (ALA scientific Instruments) on the stage of an inverted Nikon Ti2 microscope and continuously superfused for 5 min with HH buffer, fed through a valve-controlled gravity perfusion system with an approximate flow rate of 1 ml/min. The applied parameters during imaging: alternated Fura-2 excitation at 340 and 380 nm, 2 nm band-pass, 50 ms exposure) at 1 Hz using a Lambda LS Xenon lamp (Sutter Instruments, Novato, CA, USA) and a 10x/NA 0.5 objective. Emitted fluorescence was collected at 510 nm (sCMOS pco.edge camera). The ratio of fluorescence (F340:F380) was calculated. The baseline recording of F340:F380 ratio without stimulus was performed to obtain an indication of resting intracellular calcium concentration ([Ca^2+^_i_]) under continuous superfusion of HH buffer at 37 °C for 60 s. Thereafter, neurons were stimulated by the application of 25 mM KCl solution in HH buffer, superfused for 15 s, followed by superfusion with HH buffer for another 60 s to wash out KCl. 50 mM KCl was applied at the end of the recording to identify all viable, excitable cells. Only neurons responding to 50 mM KCl were included in the data analysis. Imaging was analyzed with Nikon NIS Elements software.

### Multi-electrode array recording of DRG neuron activity

DRGs were collected from C57BL/6 mice, digested and cleared of non-neuronal cells and debris as detailed in [41, 42]. Thereafter, cells were plated in 10 µl droplets (approximately 10^4^ neurons per well) of 1:1 TNB media supplemented with protein–lipid complex (Biochrom) and DMEM + 10% FBS onto six wells of a 24-well CytoView MEA plate (Axion Biosytems). After 90 min, 365 µl of media was added to each well. Four hours after plating, a 15 min recording of spontaneous activity each well was carried out on an Axion Maestro Edge at 37 °C and 5% CO_2_. Continuous data were acquired simultaneously at 12.5 kHz per electrode (16 electrodes per well). Individual spikes were detected by filtered continuous data crossing of a 6-µV static threshold. Spike number and firing rate were calculated using Axion Neural Metrics Tool. Immediately after the 4-h recording, 375 µl of MC38 cell culture medium or MC38-conditioned media was added to each well. Conditioned media were collected from 2 × 10^6^ MC38 cells grown overnight in a 10-cm tissue culture dish and centrifuged at 3900 rpm for 10 min to pellet any cellular debris. Subsequent 15-min recordings were carried out at 8 h and 20 h in vitro (4 h and 16 h of exposure to MC38 media).

### Bulk RNA sequencing from mouse DRGs

Lumbar DRG from control and MC38 tumor-bearing mice were collected after perfusion with PBS, and RNA was extracted using the RNeasy MinElute Cleanup Kit (Qiagen, Hilden, Germany), based on the manufacturer’s protocol, as described previously [44]. The prepared samples were then shipped on dry ice and the bulk RNA sequencing (mRNA library preparation) was performed by Novogene (California, USA; California Clinical Laboratory License No: 05D2146243), after ensuring the samples met or exceeded the proper quality control criteria (Agilent 2100 RNA integrity number for all samples > 7). Raw reads in FASTQ format were analyzed using the FastQC tool for quality control [45]. The STAR package was used for the reference genome mapping with the mouse genome (mm10) [46]. The gene counts were estimated from uniquely mapped reads using the feature Counts program from the Subread tool [47]. Further, the gene counts were normalized and differentially expressed genes (adjusted *p*-value < 0.05) between the control and tumor-bearing mice were calculated using the DEseq2 program [48]. The pathway enrichment analysis was performed using the IPA tool with default parameters.

### Proteome profiler antibody arrays

Sera were isolated by centrifugation of blood samples collected by terminal cardiac puncture on control and tumor-bearing mice. Serum samples were analyzed semi-quantitatively using a mouse XL Cytokine Array Kit (R&D Systems, USA) to assess circulating tumor-associated inflammatory mediator levels 1 and 3 weeks after tumor injection. To investigate factors released directly by tumor cells, conditioned media samples were generated by collecting growth media after 24 h from 10 cm dishes at 80% confluence. Samples were incubated with spotted nitrocellulose membranes according to the manufacturer’s instructions [49]. Protein concentration of samples was determined by a BCA assay prior to loading samples, to enable dilution of samples to normalize total protein content (~ 200 µg total protein). Membrane HRP luminescence intensities were visualized using an Amersham ImageQuant 800 biomolecular imager (Cytiva, USA), with an exposure time of 5 min and images were saved as high-resolution TIFF files. The registered intensity of each of the dots on the membranes was individually determined in duplicates by Image Studio software (version 5.2), and the fold-increase was calculated compared to the average value of control samples. Negative control-, and reference spots were also analyzed in each experiment to validate the analyses. Three biological replicates were measured per condition.

### Statistical analyses

All behavioral, histological and functional data are represented as mean ± standard error of mean (SEM), and all analyses were performed using GraphPad Prism 8. Differences between groups were analyzed by either two-tailed unpaired t-tests, one- or two-way analysis of variance (ANOVA) or a mixed-effects model with Sidak’s multiple comparisons test, or Mann–Whitney U-test, as described in the legends of each figure. *p* < 0.05 was considered statistically significant.

## Results

### Behavioral monitoring of the MC38 orthotopic colorectal cancer model

**Fig. 1:**
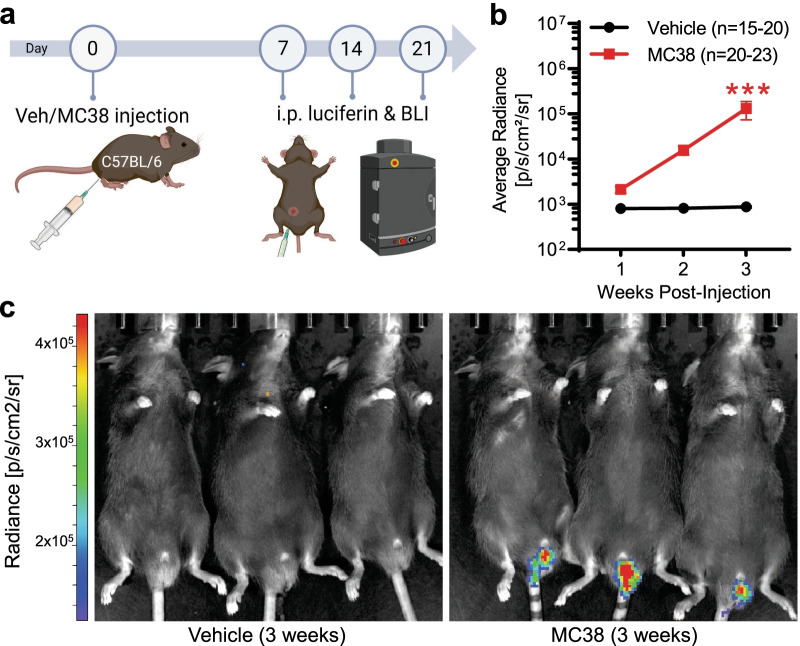
In vivo bioluminescence imaging of colon cancer development after local MC38 cell injection.** a** Schematic depicting of experimental protocol for IVIS bioluminescence measurements (animals were measured 7, 14, and 21 days after vehicle/MC38 cell injection). **b** Changes in average bioluminescence radiance values. The consistent localized increase in the measured bioluminescence signal reflects consistent tumor growth after transanal MC38 cell injection. ***: *p* < 0.001 vs MC38 week 1, mixed-effects model with Šídák's multiple comparisons test. **c** Representative image of three control (vehicle injected) and colon cancer bearing (MC38-injected) mice, 3 weeks after inoculation

**Fig. 2:**
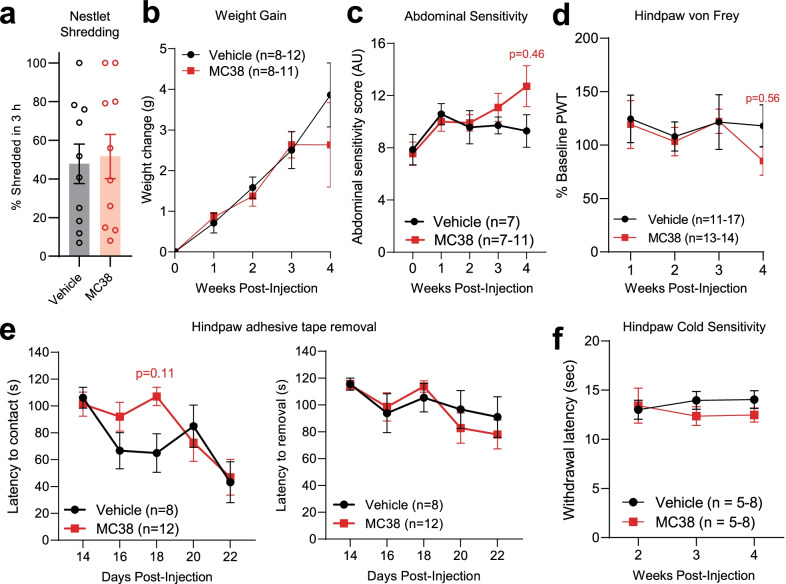
Animal well-being- and pain-related behavior tests in the MC38 colon cancer model.** a** Nestlet shredding assay. Columns depict percentage (by weight) of nestlet shredded after the 3 h experimental time period. No change in the nestlet shredding indicates proper physiological behavior (*p* = 0.8, two-tailed unpaired t test). **b** Weight gain (g) of vehicle vs MC38-injected mice after inoculation (compared to baseline at week 0, before injection). Data show no significant difference in the growth of mice with or without colon cancer (mixed-effects analysis with Šídák's multiple comparisons test). **c** Changes in abdominal sensitivity of vehicle vs MC38-injected mice after inoculation. The graph shows the scored response provoked by the application of the 0.02 g Von Frey filament to the abdomen (mixed-effects analysis with Šídák's multiple comparisons test). **d** Mechanical sensitivity of hind paws, as measured by the application of von Frey filaments, by the up-and-down method. Datapoints represent the average of percentage changes, as compared to the measured individual baseline values (measured at week 0, before inoculation). Animals did not develop significant mechanical allodynia (mixed-effects analysis with Šídák's multiple comparisons test). **e** Changes in the removal of adhesive tape from the plantar surface of the hindpaw, depicted as latency to contact (s), and latency to removal (s), respectively. Animals with colon cancer did not develop elevated or diminished tactile sensitivity (mixed-effects analysis with Šídák's multiple comparisons test). **f** Cold sensitivity of hind paws. Withdrawal threshold: time until sudden removal of the hind paw (s). The induction of colon cancer did not result in elevated cold sensitivity in the experimental period (mixed-effects analysis with Šídák's multiple comparisons test)

Following colorectal injection of MC38 cells, C57BL/6 mice were monitored weekly for tumor growth using bioluminescent imaging (BLI; Fig. 1a). MC38-injected mice developed continuously increasing luciferase signal at the site of injection, indicating increasing tumor burden over time (Fig. 1b, c)*.* Altogether, 90% of MC38-injected mice developed tumors by the 1st week after tumor inoculation (*n* = 108/120), as indicated by a robust increase in the bioluminescence signal. In order to identify tumor-associated changes in pain sensitivity and/or general well-being, MC38-injected and vehicle-injected mice were assessed for their nestlet shredding behavior and abdominal and hindpaw mechanical sensitivity, along with hindpaw cold sensitivity and nestlet shredding behavior. We found no alterations in nestlet shredding behavior at 3 weeks post-MC38 injection (*n* = 10/group), suggesting there is no debilitating pain sensitivity or disruption to spontaneous behavior at this timepoint (Fig. 2a). This observation was also underlined by no significant differences in the increase in animal weights compared to controls throughout the testing period (Fig. 2b; *n* = 8–12/group). MC38 tumor burden also did not elevate abdominal mechanical sensitivity in the first 3 weeks. However, 4 weeks after injection, MC38 tumor-bearing mice showed a strong trend toward increased sensitivity compared to vehicle-injected controls (Fig. 2c; *n* = 7–11/group). Collectively, these data suggest MC38 tumor burden does not cause overt debilitation or augment visceral pain sensitivity during the 4-week experimental period. We also did not detect any statistically significant changes in hindpaw von Frey withdrawal thresholds (Fig. 2d; *n* = 11–17/group), latency to detect/remove adhesive tape (Fig. 2e; *n* = 8–12/group), or withdrawal latencies in response to cooling (Fig. 2f; *n* = 5–8/group), suggesting that no obvious shifts in tactile or cold sensitivity are induced by MC38 tumor growth.

### MC38 colon tumor induces IENF loss

**Fig. 3:**
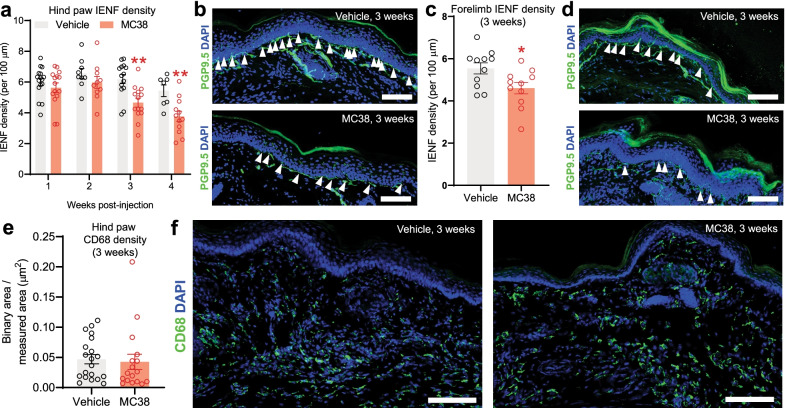
IHC assessment of hind paw and forelimb samples after MC38 injection.** a** Changes in IENF density with time in the hind paws of control vs MC38-injected mice. Colon cancer initiates a significant decrease in IENF density after the 3rd week, indicating significant neuronal damage. (**: *p* < 0.01; n = 9–13/group, two-way ANOVA with Šídák's multiple comparisons test). **b** Representative images of hind paw samples, collected 3 weeks after inoculation. White arrows mark the counted intraepidermal nerve fibers, stained with PGP9.5 antibody (green). Nuclei are stained with DAPI (blue). Scale bar: 100 μm **c** Changes in IENF density, 3 weeks after inoculation, in the forelimb samples of control vs MC38-injected mice. Similarly to the hind paw samples, our data reveal a significant decrease in IENF density, 3 weeks after local MC38 cell injection (two-tailed unpaired t test). **d** Representative images of forelimb samples, collected 3 weeks after tumor inoculation. White arrows mark the counted intraepidermal nerve fibers marked by PGP9.5 antibody. Nuclei are stained with DAPI. **e** Signal intensity of the CD68 macrophage marker in the skin of control vs MC38-injected mice, depicted as binary area/measured area. Based on the CD68 marker, there is no significant change in the number of macrophages in the epidermis, around the intraepidermal nerve fibers (*p* = 0.75, two-tailed unpaired *t* test). **f** Representative images of hind paw samples show no change in the CD68 signal intensity of the epidermis. Nuclei are stained with DAPI. Scale bar: 100 μm

Having seen no clear evidence of debilitation or sensorimotor dysfunction in MC38-injected mice, we next assessed MC38-induced changes to IENF density in the plantar skin, a prominent symptoms of peripheral neuronal damage [37, 39]. Plantar punch biopsies of hindpaws from tumor-bearing and control animals (*n* = 7–16 samples/group from ≥ 3 biological replicates) were analyzed by immunohistochemistry weekly after MC38 cell injection. We found no change in hindpaw IENF density (nerve endings/100 µm of epidermis) in the first 2 weeks post-injection (Fig. 3a). However, at 3 and 4 weeks, there was a significant decrease in IENF density in tumor-bearing animals compared to vehicle-injected controls (Fig. 3b). To investigate whether this phenomenon was systemic, or restricted only to sensory innervation that overlaps with the thoracolumbar (T10–L1) and lumbosacral (L6–S1) innervation of the colon/rectum [50], we also analyzed forepaw skin, which is innervated by levels C6–T2, and as such does not innervate the colon/rectum [51]. We also found a significant decrease in IENF density in forepaw skin at the third week of MC38 tumor development (Fig. 3c, d; *n* = 11/group from ≥ 2 biological replicates), indicating that reduced IENF density is not confined to those ganglia that also provide sensory innervation to the colon. This significant alteration suggested that peripheral nerve endings experience injury/damage in MC38-injected mice, despite finding no significant changes in mouse well-being or pain-related behaviors. Since prior studies of nerve injury have reported increased macrophage density in the skin [21], we determined macrophage density in skin biopsies three weeks after MC38 injection, using the macrophage marker CD68. We found no alterations in CD68 density in the hind paw skin of tumor-bearing mice (Fig. 3e, f; *n* = 17–20/group from ≥ 3 biological replicates), indicating that the IENF loss associated with MC38 injection does not elevate skin macrophage density.

### MC38 tumor does not change neuronal damage-, or macrophage marker intensity in mouse DRG

**Fig. 4:**
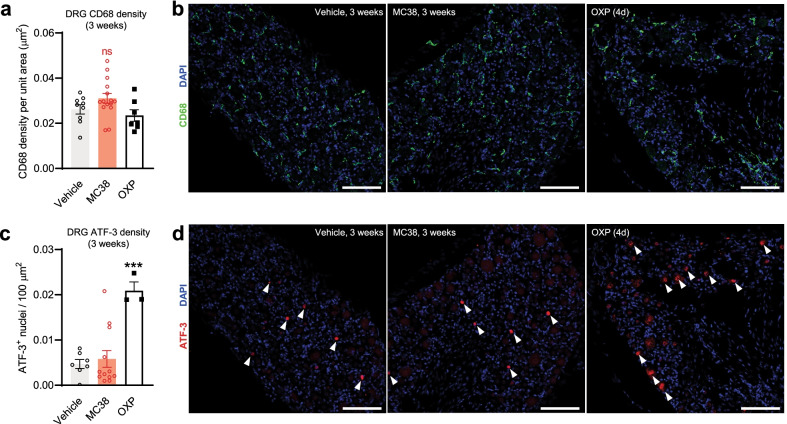
IHC assessment of the neuronal damage marker ATF-3 in DRGs. Lumbar DRG samples were collected 3 weeks after tumor inoculation. Naïve mice were treated with oxaliplatin (OXP) as a positive control for ATF-3 activation. **a**, **b** No change in the signal intensity of the macrophage marker CD68 (vehicle vs MC38-injected or oxaliplatin-treated mice). **c**, **d** Representative images show no change in ATF-3 signal (samples from a vehicle vs an MC38-injected mouse, collected 3 weeks after injection). However, oxaliplatin treatment alone (positive control; cumulative dose: 12 mg/kg i.p, in 3 days) causes neuronal damage, hence significantly elevating ATF-3 signal density. White arrows indicate cells positive for ATF-3. Scale bar: 100 μm. *** = *p* < 0.001, one-way ANOVA, Tukey’s multiple comparisons test

Since MC38 tumor growth was associated with systemic reductions in IENF density, we investigated well-established indicators of neuronal damage at the level of the DRG: elevated macrophage density and increased expression of ATF-3 [52–54]. We did not observe any significant changes in lumbar DRG CD68 density or ATF-3 expression in samples from tumor-bearing vs control mice (Fig. 4a–d; *n* = 7–12/group from ≥ 3 biological replicates). In contrast, repeat dosing with oxaliplatin (cumulative dose: 12 mg/kg i.p.; *n* = 3) as a positive control did significantly elevate ATF-3 expression (Fig. 4c, d). This suggests that MC38 tumor-induced IENF loss is not associated with conventional markers of neuronal damage in the DRG.

### MC38 colon tumor induces deficits in DRG mitochondrial function, calcium flux and electrical activity

**Fig. 5:**
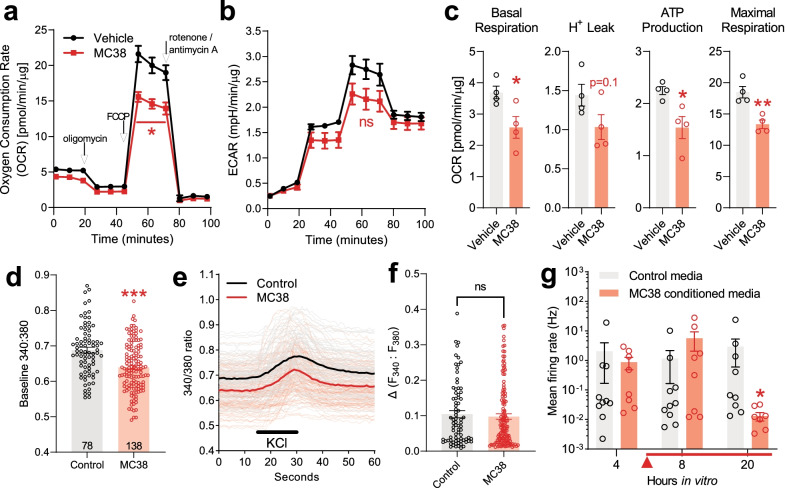
Mitochondrial function, Ca^2+^ homeostasis and spontaneous activity of lumbar DRG neurons. **a**–**c** Seahorse mitochondrial function assay on DRGs collected 3 weeks after inoculation (*n* = 3 biological replicates/group). All values are normalized to mitochondrial-dependent respiration and the protein load. **a**, **b** Basal oxygen consumption rate (OCR) and extracellular acidification rate (ECAR) are measured prior to treatment with oligomycin (complex V inhibitor) to calculate proton leak and ATP production (**c**). Maximal respiration is measured post-addition of the uncoupler FCCP. Values post-rotenone/antimycin A (complex I/III inhibitors) reflect non-mitochondrial oxygen consumption. This ‘background’ oxygen consumption is subtracted from the values in **c**. *: *p* < 0.05; **: *p* < 0.01, two-tailed unpaired *t* test. **d**, **e** Live-cell Ca^2+^ imaging on DRGs collected 3 weeks after MC38 inoculation. Number of measured cells: *n* = 78 and *n* = 138 for the vehicle and MC38 groups, respectively, from 2 biological replicates/group. Lumbar and non-lumbar DRG neurons were cultured and tested separately for extra control (additional Fig. 1). **d** DRGs from MC38-injected mice show significantly lower resting/baseline [Ca^2+^]_i_ compared to vehicle-injected mice. The columns depict the average values of the 60-s baseline measurements for each group (***: *p* < 0.001, two-tailed unpaired *t* test). **e** Traces (average traces in bold) showing changes in intracellular [Ca2 +]i after superfusion of 25 mM potassium chloride (KCl) for 15 s. **f** No significant change in F340:F380 amplitude induced by KCl in **e** (two-tailed unpaired *t* test). **g** Multielectrode array analysis of DRG neurons from naïve mice with or without the addition of MC38-conditioned media (*n* = 8–10 wells/group, from at least 3 different cell cultures). Neuronal activity was measured every 4 h, for 24 h. Conditioned media or control solution (unconditioned MC38 media) was added after the 4 h measurement (marked with a red arrow and line). The addition of MC38-conditioned media was associated with a significant decrease in the spontaneous activity of DRG neurons at 20 h in vitro (*: *p* < 0.05 vs control, Mann–Whitney U test)

A well-described mechanism of neuropathic pain is mitochondrial dysfunction in sensory neurons [55]. Therefore, we tested whether MC38 tumor growth was associated with mitochondrial dysfunction in mouse DRGs. Despite not detecting elevated ATF-3 expression or CD68^+^ macrophage density, MC38 tumor development led to significant mitochondrial dysfunction in the DRGs of tumor-bearing animals, by the third week after MC38 injection (Fig. 5a–c; *n* = 4 biological replicates/group in duplicates). This is indicated by significant changes in the oxygen consumption rate (OCR; Fig. 5a); the extracellular acidification rate (ECAR) approached, but did not reach statistical significance (Fig. 5b). Further analysis revealed reduced basal and maximal respiratory capacity and ATP production in DRG cultures from MC38 mice (Fig. 5c). Importantly, these effects were observed on DRGs pooled from every level between C4 and L5, again suggesting this phenomenon is unlikely to be confined to those DRG neurons innervating the colon.

Since mitochondrial function is a major contributor to calcium dynamics in DRG neurons [56], we next performed live-cell calcium imaging of DRGs from control and MC38 tumor-bearing mice (Fig. 5d, e; *n* = 78–138 cells/group from ≥ 3 biological replicates). DRG neurons from MC38 tumor-bearing mice showed a small but significant decrease in baseline 340:380 nm Fura-2 ratio (Fig. 5d), indicating decreased resting [Ca^2+^_i_] levels. Lumbar and non-lumbar DRG neurons were cultured and tested separately (data not shown), but, since no differences were observed (similar [Ca^2+^_i_] decrease at both the lumbar and upper levels, see Additional file 1: Fig. S1), the data were pooled. Despite this decrease in baseline [Ca^2+^_i_], the response to a depolarizing stimulus (25 mM KCl) [43] did not show any difference in response amplitude (Fig. 5e). Finally, we wanted to establish whether exposure to MC38-derived factors could be altering DRG neuron activity. Naïve C57BL/6 DRG neurons were dissociated and cultured on multi-electrode array plates (*n* = 8–10 cell culture wells/group from ≥ biological replicates). Four hours after plating, 50% of growth media was exchanged for fresh MC38 media (‘control media’) or media in which MC38 cells had been grown for 48 h (‘MC38-conditioned media’). At 20 h in vitro, DRG neurons exposed to MC38-conditioned media displayed a significantly lower spontaneous firing rate (Fig. 5f). Collectively, this suggests that MC38-derived factors can influence DRG mitochondrial function and calcium homeostasis, which may underlie the reduced activity of cultured DRG neurons.

### MC38 tumor growth is associated with inflammatory mediator production

**Fig. 6:**
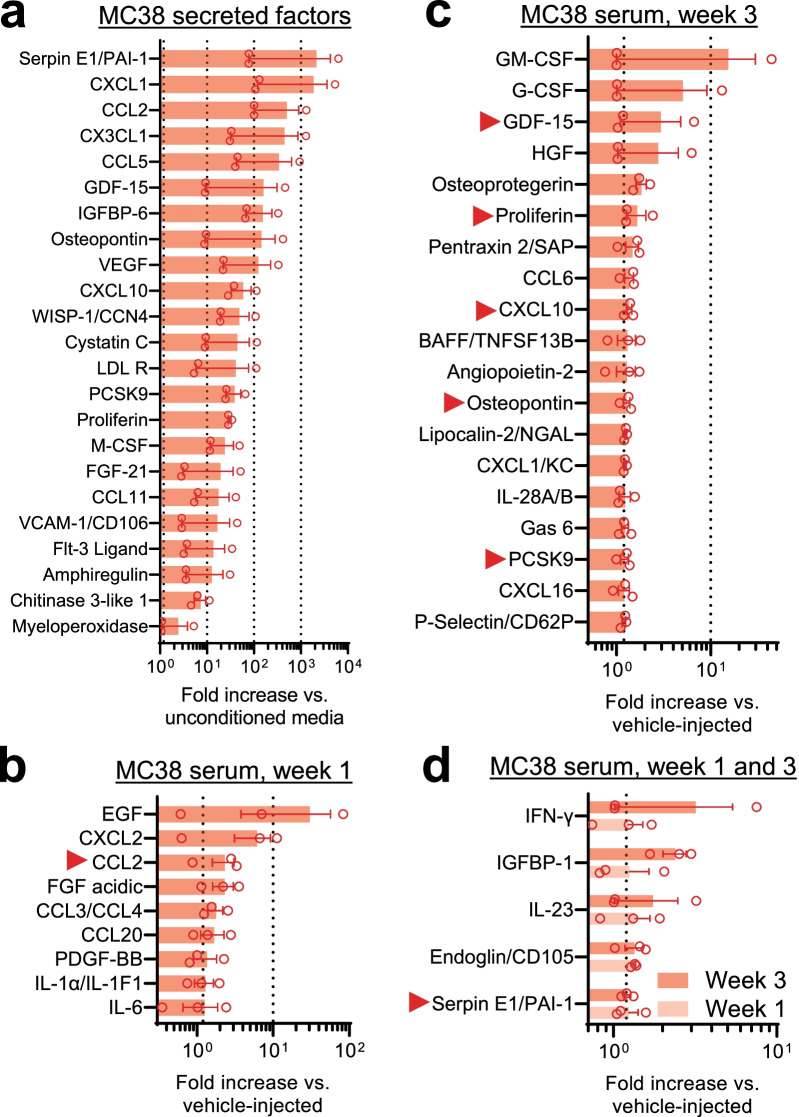
Factors secreted by MC38 cells and inflammatory changes in MC38 tumor-bearing mice. The change in different inflammatory mediators was measured by a complex proteome profiler array and presented as fold-change in signal intensity compared to the average of given control values (*n* = 3/group, in duplicates). **a** Inflammatory mediators found to be secreted by MC38 cells (factors with elevated signal intensity in conditioned media vs un-conditioned media, as presented by fold-increase). **b**–**d** Elevated inflammatory mediators in the plasma samples of MC38-injected mice compared to control (vehicle injected) mice. **b** Increased factors in blood samples collected 1 week after MC38 inoculation. **c** Increased factors in blood samples collected 3 weeks after MC38 inoculation. **d** Factors increased both 1 and 3 weeks after tumor cell injection. Red arrows mark mediators found to be secreted by MC38 cells, as well as increased in the collected plasma samples

In order to assess the secreted factors that could be driving MC38-induced DRG neuron dysfunction, we analyzed MC38 secretory output using Proteome Profiler assays (Mouse XL Cytokine Array Kit; R&D Systems [49]). Culture media in which MC38 cells were grown (2 × 10^6^ cells, 48 h) was compared with uncultured media (*n* = 3 different MC38 cell cultures in duplicates). Factors showing ≥ 1.2-fold increase compared to control are depicted in Fig. 6a. An additional table shows all measured cytokines (see Additional file 2: Tables S2 and S3). Abundant secretion of chemokines (CXCL1, CCL2, CX3CL1, CCL5, CXCL10, CCL11) and growth factors (GDF-15, VEGF, M-CSF, FGF-21, amphiregulin) was detected in MC38-conditioned media. We also collected serum from MC38 tumor-bearing mice and vehicle-injected controls, 1 week and 3 weeks after MC38 injection (n = 3 biological replicates/group in duplicates). At week 1, elevated levels of cytokines (IL-1α, IL-6), chemokines (CXCL2, CCL2, CCL3/4, CCL20) and growth factors (EGF, FGF acidic, PDGF-BB) were detected (Fig. 6b). 3 weeks after MC38 injection, the following mediators were shown to be increased by more than a 1.2-fold (in decreasing order): GM-CSF, G-CSF, GDF-15, HGF, Osteoprotegerin, IL-23, Proliferin, Pentraxin-2, CCL6, CXCL10, BAFF, Angiopoietin-2, Osteopontin, Lipocalin-2, CXCL1, IL-28A, Gas6, Proprotein Convertase 9, Serpin E1, CXCL16, P-Selectin (Fig. 6c). IFN-γ, IGFBP-1, IL-23, Endoglin and Serpin E1 were elevated in tumor-bearing serum at both timepoints (Fig. 6d). Altogether, seven factors were detected both in MC38-conditioned media and MC38 tumor-bearing serum: CCL2, GDF-15, Proliferin, CXCL10, Osteopontin, PCSK9 and Serpin E1. In agreement with our data, the chemokines shown to be increased here were also found to be elevated in different previous studies investigating cancer or neuropathic pain. Given their extensive role in the development of different neuropathies (including CIPN [57]), it is plausible that some of these factors play a significant part in the neuropathy observed here as well.

### MC38 tumors induce transcriptional changes in the DRG

**Fig. 7:**
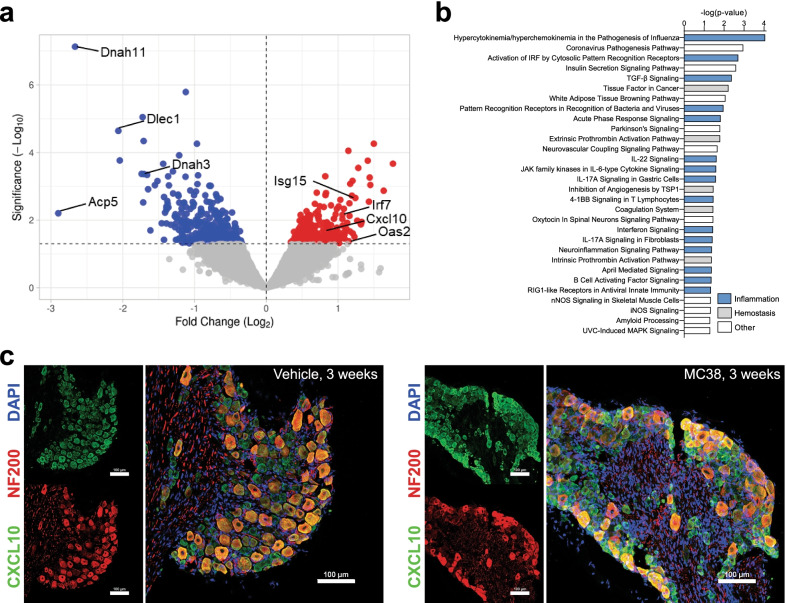
Bulk RNA sequencing of DRGs from MC38-injected mice. Bulk RNA sequencing analysis on lumbar DRGs of mice collected 3 weeks after tumor inoculation (*n* = 3/group). **a** Volcano plot showing the different genes with significantly decreased (blue) or increased (red) expression (*p*≤ 0.05). **b** Gene expression pathway enrichment analysis (MC38-injected vs vehicle-injected mice). Blue: immune/inflammation-related pathways; grey: hemostasis/clotting/thrombotic pathways (consistent with elevation of serpin E1 and chitinase-3 like 1, among others, in the proteome profiler arrays). **c** Immunohistochemistry of lumbar DRG from vehicle-injected and MC38 tumor-bearing mice at 3 weeks indicates CXCL10 immunoreactivity (green) overlaps with the neuronal marker NF200 (red), as well as a subpopulation of small–medium diameter neurons. DAPI: blue, scale bar = 100 μm

In order to better understand the signaling events and gene expression changes that MC38 tumors may be inducing to elicit functional changes in neuronal physiology, we subjected lumbar DRGs from MC38 cancer cell injected mice and control (*n* = 3/group) to bulk RNA-sequencing (Fig. 7). 134 differentially expressed genes were identified, 39 of which were significantly upregulated (*p* < 0.05; Fig. 7a and Additional file 1: Table S1). Functional and disease enrichment analysis on upregulated genes from the tumor-bearing mice showed cancer as a top enrichment (number of genes: 23, see Additional file 4: Fig. S2a, b). Consistent with the elevated levels of multiple cytokines and chemokines in tumor-bearing serum, the pathway analysis showed the most robust increase in the ‘Role of hypercytokinemia/hyperchemokinemia in the Pathogenesis of Influenza’ pathway, along with numerous other inflammation-related pathways (Fig. 7b). Several pathways related to thrombosis/coagulation (hemostasis) are also consistent with the hypercoagulable state attributed to colon cancer [58]. Collectively, these data indicate that the tumor induces transcriptional changes due to systemic inflammation. Of particular note is the significant upregulation of CXCL10 expression (Log_2_ fold increase: 0.843, *p* = 0.012). This increased expression in bulk RNA is likely derived from DRG neuron somata, as they are the chief CXCL10-immunoreactive cell type in immunohistochemistry (Fig. 7c). It is notable that the proteome profile data also identified elevated expression of this chemokine in MC38 tumor-bearing mouse serum.

### CT26 orthotopic colon cancer model in BALB/c mice

**Fig. 8:**
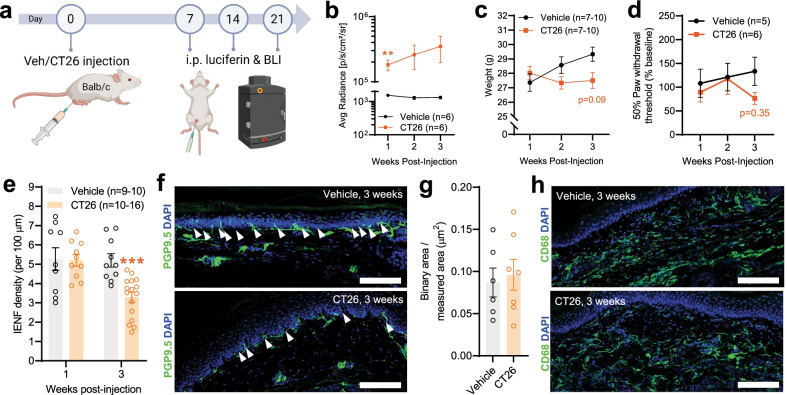
Tumor development, behavioral-, and IENF density changes in the CT26 colon cancer model. The data from animals with local injection of CT26 cells reveal very similar changes and patterns to the MC38 model. **a** Schematic representation of the experimental protocol for the IVIS in vivo bioluminescence measurements (animals were measured 7, 14, and 21 days after vehicle/CT26 cell injection). The consistent robust increase in the bioluminescence signal is depicted in **b** and indicates persistent colon cancer development (**: *p* < 0.01 vs vehicle-treated, two-way ANOVA with Šídák's multiple comparisons test). **c** No significant change in the development (animal weight, g) of mice with or without CT26 initiated colon cancer (mixed-effects analysis with Šídák's multiple comparisons test). **d** Von Frey testing of hind paws revealed no significant alteration in mechanical sensitivity. Datapoints represent the average of percentage changes, compared to the measured individual baseline values (measured at week 0, before inoculation. Two-way ANOVA with Šídák's multiple comparisons test). **e–h** Immunohistochemical analysis of hind paw samples: changes in IENF (PGP9.5) and macrophage (CD68) signals. **e** Changes in IENF density with time in hind paws of control vs CT26-injected mice. The IENF density in the hind paws of CT26-injected mice was significantly decreased compared to control (vehicle inj.), 3 weeks after tumor inoculation (***: *p* < 0.001, two-way ANOVA with Šídák's multiple comparisons test). **f** Representative images show the decrease in IENF density compared to control, 3 weeks after tumor inoculation. White arrows indicate intraepidermal nerve fibers. Scale bar: 100 μm. **g** No significant changes in CD68 signal intensity at the 3rd week in the hind paws of control vs CT26-injected mice (*p* = 0.73, two-tailed unpaired *t* test). **h** Representative images of hind paw samples show no change in the CD68 signal intensity of the epidermis (3 weeks after cell injection). Scale bar: 100 μm

**Fig. 9:**
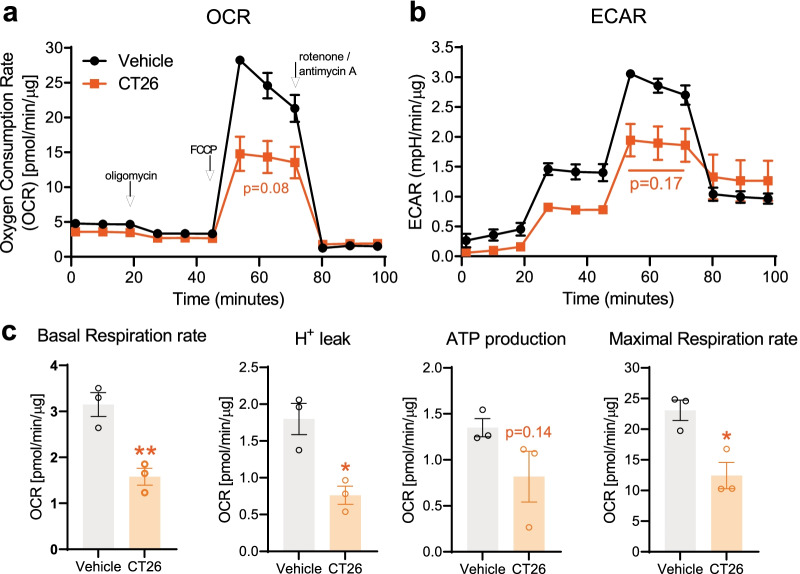
Seahorse assay of DRG cells in the CT26 model. The mitochondrial function assay—performed on living lumbar DRGs of control and colon cancer bearing mice—reveals significant mitochondrial dysfunction 3 weeks after CT26 cell injection (similarly to the MC38 model). All values are normalized to mitochondrial-dependent respiration and the protein load. **a, b** Trend to reduced OCR and ECAR after addition of the uncoupler FCCP. **c** Deficits in mitochondrial function in CT26 versus vehicle-injected mice become statistically significant when corrected for non-mitochondrial oxygen consumption. *: *p* < 0.05; **: p < 0.01, two-tailed unpaired t test

We next set out to determine if the disruption of DRG neuron function seen in MC38 tumor-bearing mice would generalize to other mouse strains and cancer cell lines. Similar to the MC38 model, we carried out orthotopic injection of luciferase-expressing CT26 cells into Balb/c mice, followed in vivo by bioluminescence measurements (Fig. 8a). All mice injected with CT26 cells developed colon tumors (*n* = 16). When investigating the weekly tumor growth in experimental animals, we found a significant increase in bioluminescence signal at the 1st week after cell injection, which continuously further elevated during the 3-week time period (Fig. 8b; n = 6/group), indicating the development of colon cancer. Again, similarly to the MC38 model, we found no significant differences in animal weight (Fig. 8c; *n* = 7–10/group). Mechanical sensitivity of mice was assessed 1, 2 and 3 weeks post-tumor injection. Similar to the MC38 model, we found no significant change in hindpaw mechanical sensitivity throughout the 3-week time period (Fig. 8d; *n* = 5–6/group). Crucially, 3 weeks after CT26 tumor cell injection, a significant decrease in the IENF density of samples from the tumor group was observed (Fig. 8e, f; *n* = 9–16 samples/group from at least 3 biological replicates), without any change in macrophage density (Fig. 8 g, h; *n* = 6–7/group from at least 2 biological replicates), mirroring the sensory neuron dysfunction seen in C57BL/6 mice with MC38 cells. This IENF loss was again accompanied by mitochondrial dysfunction in the DRGs of tumor-bearing animals, as indicated by significant changes in both OCR and ECAR (Fig. 9a–c; *n* = 3 biological replicates/group in duplicates).

### CT26 tumor-induced inflammatory changes

**Fig. 10:**
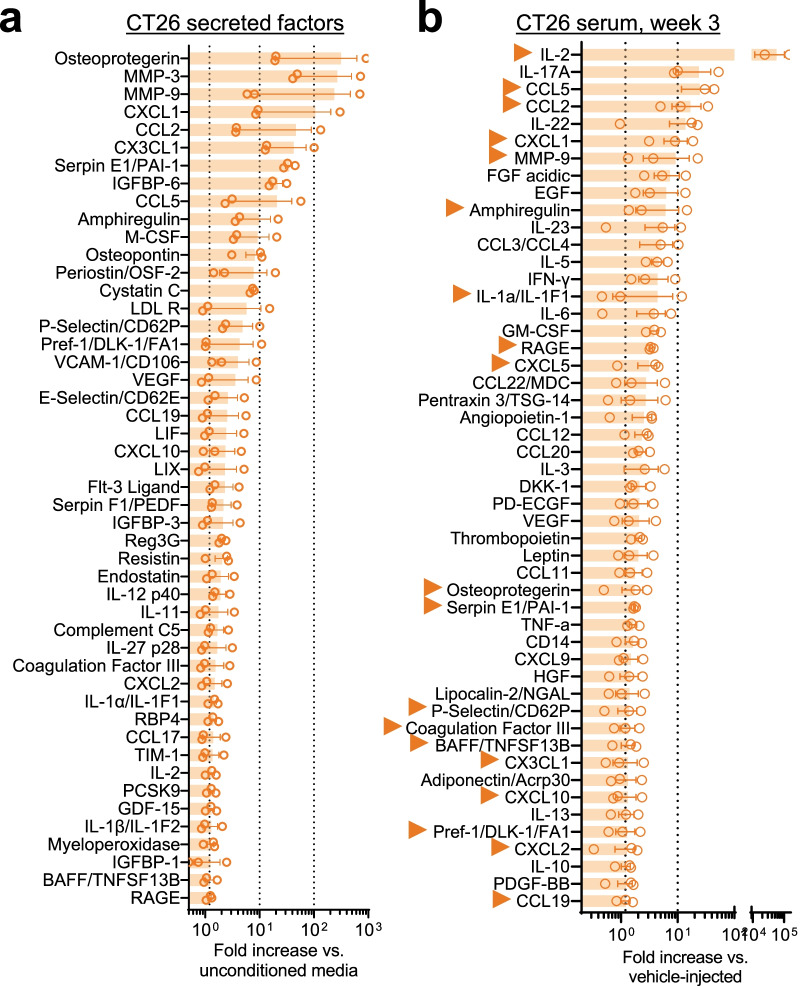
Factors secreted by CT26 cells and inflammatory changes in CT26 tumor-bearing mice. The change in different inflammatory mediators was measured by proteome profiler array and presented as fold-change in signal intensity compared to mean control values (*n* = 3/group, in duplicates). **a** Inflammatory mediators found to be secreted by CT26 cells (factors with elevated signal intensity in conditioned media vs un-conditioned media, as presented by fold-increase). **b** Increased factors in blood samples collected 3 weeks after CT26 inoculation. Arrows mark mediators detected in CT26-injected mouse serum that were also detected in CT26-conditioned media (**a**)

To investigate the tumor-induced inflammatory alterations in BALB/c mice, a cytokine array experiment was performed on mouse plasma samples 3 weeks after CT26 tumor cell injection. An additional table shows all measured cytokines (see Additional file 2: Tables S2 and S3). Similar to the MC38 model, we found robust alterations in secreted factor output in CT26-conditioned media (Fig. 10a; *n* = 3 cell culture replicates). The following mediators showed at least a 1.2-fold signal increase compared to un-conditioned media samples: cytokines (IL-1α/β, IL-2, IL-11, IL-12 p40, IL-27 p28, LIF,) chemokines (CXCL1, CXCL2, CCL2, CX3CL1, CCL5, CCL17, CCL19, CXCL10, LIX) and growth factors (Osteoprotegerin, IGFBP-1/3/6, M-CSF, VEGF) predominate (Fig. 10a; *n* = 3 biological replicates/group in duplicates). In serum of CT26 tumor-bearing mice, there was extensive overlap with the factors detected in CT26-conditioned media: following mediators identified in conditioned media showed at least a 1.2-fold increase in CT26 tumor-bearing mice: IL-1α, IL-2, IL-13, CCL2/5/19, LIX, CX3CL1, CXCL1/2/10 and CCL19 (Fig. 10b).

Across serum, conditioned media and both tumor types, four factors are consistently detected: CCL2, CXCL1, CXCL10 and Serpin E1. Collectively, these data indicate that similar pro-inflammatory pathways across two colorectal cancer models are associated with subclinical mitochondrial dysfunction and IENF loss in sensory neurons.

## Discussion

With improved treatment of colorectal cancer comes the potential for long-term side effects in survivors, a population that is set to continue growing for the foreseeable future [59]. Although clinical data highlight peripheral neuropathy as a major side-effect in cancer survivors [60] and the fact that the pro-inflammatory milieu generated by tumors can affect neuronal function [4, 6], the extent to which colorectal cancer is directly responsible for neuropathy symptoms remains under-explored. Our study demonstrates colon tumor-initiated neuronal damage in a preclinical setting, by the application of orthotopic colon cancer models in two different mouse strains. Inoculation of both MC38 and CT26 cancer cells led to continuous tumor development without overt changes in overall activity, shifts in pain sensitivity or cachexia (Figs. 2, 8c, d). Only 4 weeks post-tumor injection did MC38 tumor-bearing mice begin to develop a trend toward abdominal hypersensitivity and reduced/stalled weight gain (Fig. 2b). The most typical symptoms of neuropathic pain, cold and mechanical hypersensitivity [61] were also unaffected by tumor growth (Figs. 2d–f, 8d).

Despite the lack of behavioral symptoms consistent with neuropathy, we assessed cutaneous IENF density, another typical indicator of peripheral neuropathy [62, 63]. Damage of peripheral neurons was detected as decreased IENF density in hindpaw skin of tumor-bearing mice 3 weeks after cancer cell injection. Furthermore, a similar reduction in forepaw skin IENF density suggests that tumor growth is associated with systemic reductions in IENF density. Since the dermatomes of the forepaw are innervated by ganglia outside of the thoracolumbar innervation of the lower GI tract [50], injury of DRG neurons that innervate both the colorectum and epidermis seems highly unlikely. Rather, such changes would be consistent with a systemic change in circulating factors eliciting neuronal dysfunction. Crucially, colon cancer patients commonly show symptoms of peripheral neuronal damage when tested (IENF density loss; minor sensory deficits in the extremities), despite not presenting with overt symptoms of pain or sensory loss [60, 63]. That said, it is important to underline that there is not always a direct association of IENF loss with neuropathic symptoms, and further work is needed to understand the degree to which tumor-induced neuropathy modifies lifetime risk of neuropathic pain [63, 64].

Consistent with the lack of alteration in pain behaviors, we did not detect increased macrophage density [52] or ATF-3 expression in the DRG [65] of tumor-bearing mice, which contrasts with the changes seen following induction of CIPN or traumatic nerve injury. Finally, one of the main factors underlying neuropathy and neuropathic pain is mitochondrial dysfunction [17, 55, 66]. The Seahorse assay revealed substantial mitochondrial dysfunction in tumor-bearing mice, indicated by both OCR and ECAR (Figs. 5a–c, 9). Deficits of this magnitude (approximately 30% reduction in OCR) are less severe than those seen in models of cisplatin-induced CIPN (typically 50–60% reduction in OCR), changes that are associated with significant pain hypersensitivity and IENF loss [28]. This suggests that the relationship between mitochondrial dysfunction and pain hypersensitivity may be non-linear, that mitochondrial dysfunction occurs prior to the development of pain, or that mitochondrial dysfunction induced by tumor growth does not contribute to pain hypersensitivity. At this point, we cannot rule out mitochondrial dysfunction/reduced ATP production in tissues outside the DRG. Indeed, such effects have been reported in the skeletal muscle of patients with cancer [67, 68], though energetic demands and post-mitotic nature of neurons makes them particularly vulnerable [69, 70]. As such, our data are consistent with observations that energy availability and metabolic derangement are common features of cancer and cancer-related fatigue [71–73].

To further address tumor-induced neuronal dysfunction, calcium imaging was performed on DRG neurons. Reduced intracellular Ca^2+^ levels ([Ca^2+^_i_]) were recorded in DRG neurons from tumor-bearing mice. Though this can appear counterintuitive when compared with chronic pain states which tend to show hyperexcitability [74], this finding is consistent with prior reports showing low [Ca^2+^]_i_ after neuronal damage [75–79]. For example, Andreas Fuchs and co-workers showed that spinal nerve ligation decreased resting [Ca^2+^]_i_ in rat DRGs [77]. Reduced neuronal [Ca^2+^]_i_ is known to precipitate cell loss, a feature in different forms of neuropathy [77, 80], However, it is unclear as yet if reduced [Ca^2+^_i_] is directly tied to neuropathy and IENF loss, or whether this is indicative of a systemic hypocalcemic state, as has been reported for hematological and colorectal cancers [81].

Mitochondria play a prominent role in in neuronal Ca^2+^ signaling [82], and abnormal mitochondrial function can lead to axonal degeneration as well as disturbances in Ca^2+^ homeostasis, which can manifest in low [Ca^2+^_i_] levels and neuronal damage [83]. The specific contribution of plasma membrane and organelle Ca^2+^ pumps, such as the sarco-endoplasmic reticulum Ca^2+^-ATPase (SERCA), should be investigated, since they may contribute to reduced [Ca^2+^_i_] levels, mitochondrial dysfunction and/or ER stress [78]). In summary, to the outwardly asymptomatic, but prominent neuronal dysfunction induced by tumor growth may have implications for any future neurological insults incurred, as a side-effect of cancer treatment for example.

In search of circulating factors that could underlie systemic sensory neuron dysfunction, cytokine arrays of tumor-bearing and control mouse plasma revealed systemic inflammatory changes (Figs. 6, 10). Interferon-γ was notable for its detection both 1 week and 3 weeks after tumor injection. Several chemokines (CCL2, CXCL1, CXCL2, CXCL10) were also increased in MC38 and CT26-conditioned media and tumor-bearing mouse serum. Interestingly, many chemokines are classed as ‘interferon-stimulated genes’ [84], an assertion borne out by our DRG RNA sequencing data and consistent with the significant increase in genes pertaining to the ‘hypercytokinemia/hyperchemokinemia’ process (Fig. 7). Prior studies found increased chemokine expression in tissues from colorectal cancer patients was associated with disease progression [85, 86].

The other main pathways highlighted by the RNAseq data relate to hemostasis/hypercoagulability (e.g., ‘role of tissue factor in cancer,’ ‘intrinsic/extrinsic prothrombin activation pathway,’ ‘coagulation system’). Colorectal cancer is often associated with hypercoagulability, to the extent it substantially increases the risk of thrombosis [58]. It is plausible that impaired endoneurial blood flow results from such clotting events, contributing to mitochondrial dysfunction and neuropathy, though this needs to be addressed experimentally.

Our bulk RNA sequencing data on DRGs from MC38 tumor-bearing mice also revealed a significant increase in the expression of CXCL10 (Fig. 7). Literature data indicate that the expression of chemokines and their receptors, such as CCL2/CCR2, CXCL1/CXCR2, (among others) are altered in CIPN [57]. CXCL2 was shown to promote neuropathic pain in a recent study of trigeminal neuropathic pain [87]. Several studies also suggest a key role of CXCL10/CXCR3 signaling in neuropathy [88–90]. The extent to which DRG inflammation is a cause or consequence of DRG mitochondrial dysfunction remains to be established. NLRP3 inflammasome activation and production of inflammatory mediators could be downstream of ROS production from dysfunctional mitochondria [91], or inflammatory mediator signals originating from the circulation could be responsible for inducing DRG mitochondrial dysfunction.

It is well-established that pre-existing neuropathy of various etiologies is a risk factor for subsequent development of neuropathic pain [17]. In this context, tumor-induced neuropathy may have major implications for CIPN. Oxaliplatin treatment is still a cornerstone of colon cancer therapy. However, a subset of patients (about 40%) develop chronic, intractable CIPN symptoms (such as mechanical allodynia or cold hypersensitivity) [17]. Based on our observations, tumor-induced inflammation may represent a crucial risk factor in chronic CIPN development, which should be further investigated in the future.

The current study focuses on effects in male mice, since colorectal cancer is more common in men [2] and because we chose to increase the generalizability of our findings, by employing the same model on a different genetic background, engrafting CT26 cells into BALB/c mice. Future studies will explore the effects of tumor growth in female mice. Without significant alterations in behavior, the CT26 model showed a similar decrease in IENF density, as well as indicators of mitochondrial dysfunction in DRG neurons (at the same time point as in the MC38 model—3 weeks after engraftment, Fig. 8). The proteome profiler arrays of serum 3 weeks after engraftment depicted a similarly robust systemic inflammation, with substantial overlap with the MC38 model (i.e., chemokines), as described above (Fig. 10).

It remains to be seen if the pro-inflammatory and hypercoagulable state associated with other cancer types elicits similar neuronal dysfunction, but subclinical peripheral neuropathy is also known to occur in patients with lung cancer or multiple myeloma [15, 92], suggesting this phenomenon may extend beyond colorectal cancer. Our observations indicate that the tumor-induced systemic changes include peripheral neuronal dysfunction, without any pharmacological or other intervention. The increased mediators might be crucial in the induction of chronic, intractable CIPN, developing in a subset of colon cancer patients. This and the exact mechanism by which colon tumor induces systemic, peripheral neuronal dysfunction in mice warrants further investigation in future studies.

## Conclusions

Our study, conducted on two orthotopic colon cancer models of different genetic backgrounds, reveals that the development of colon cancer in mice leads to significant peripheral neuronal dysfunction, without prominent behavioral alterations. The observed neuronal changes are presumably induced by the prominent and complex inflammatory processes induced by tumor growth. The chemokines CCL2, CXCL1, CXCL2, and particularly CXCL10 might be key factors at play.

## Supplementary Information

**Additional file 1: Table S1.** Additional table listing all differentially expressed genes in MC38 tumor-bearing versus vehicle control dorsal root ganglia.

**Additional file 2: Tables S2 and S3.** Additional tables listing all detected inflammatory mediators in cancer cell growth medium and tumor-bearing mouse serum.

**Additional file 3: Figure S1.** Additional figure showing the results of live-cell Ca^2+^ imaging, presenting the separately analyzed DRG neurons isolated from lumbar **a**–**c** and non-lumbar (**d**–**f**). A similar decrease in [Ca^2+^]_i_ was observed in DRG neurons from both levels in tumor-bearing mice compared to control, 3 weeks after tumor inoculation.

**Additional file 4: Figure S2.** Additional figure showing disease and functional enrichment analysis of RNA sequencing of DRGs from MC38-injected mice.

## Data Availability

The RNA sequencing data in this study are deposited in the BioProject database under ID PRJNA857898. All other datasets generated during the current study are available from the corresponding author on reasonable request.

## Abbreviations

ATF-3: Activating transcription factor 3
BAFF: B-cell activating factor/TNFSF13B
BCA: Bicinchoninic acid
BSA: Bovine serum albumin
CD14/68: Cluster of differentiation 14/68
CIPN: Chemotherapy-induced peripheral neuropathy
CXCL1/2/5/9/10/16: C-X-C chemokine ligand 1/2/5/9/10/16
CCL2/3/4/5/6/11/12/17/19/20/22: C–C chemokine ligand 2/3/4/5/6/11/12/17/19/20/22
CMV: Cytomegalovirus
CX3CL1: C-X3-C chemokine ligand 1/fractalkine
DAPI: 4′,6-Diamidino-2-phenylindole
DKK-1: Dickkopf WNT signaling pathway inhibitor 1
DRG: Dorsal root ganglion
ECAR: Extracellular acidification rate
EDTA: Ethylenediaminetetraacetic acid
EGF: Epidermal growth factor
FBS: Fetal bovine serum
FCCP: Carbonyl cyanide-p-trifluoromethoxyphenylhydrazone
FGF/-21: Fibroblast growth factor/-21
Flt-3: Fms-like tyrosine kinase 3/CD135
Gas6: Growth arrest specific 6
G-CSF: Granulocyte colony-stimulating factor
GDF-15: Growth/differentiation factor-15
GM-CSF: Granulocyte–macrophage colony-stimulating factor
HEPES: 4-(2-Hydroxyethyl)-1-piperazineethanesulfonic acid
HGF: Hepatocyte growth factor
HH: HEPES-buffered Hanks’ balanced salt solution
HRP: Horseradish peroxidase
IENF: Intra-epidermal nerve fiber
IFN-γ: Interferon-gamma
IGFBP-1/3/6: Insulin-like growth factor-binding protein-1/3/6
IL-1α/1β/2/3/5/6/10/11/12p40: Interleukin-1α/1β/2/3/5/6/10/11/12p40/13/17A/22/23/27p28/28A/13/17A/22/23/27p28/28A
IPA: Ingenuity pathway analysis
LDL-R: Low density lipoprotein receptor
LIF: Leukemia inhibitory factor
M-CSF: Macrophage colony-stimulating factor
MMP-3/9: Matrix metallopeptidase-3/9
NF200: Neurofilament 200
OCR: Oxygen consumption rate
PB: Phosphate buffer
PBS: Phosphate-buffered saline
PCSK9: Proprotein convertase subtilisin/kexin type 9
PD-ECGF: Platelet-derived endothelial cell growth factor
PDGF-BB: Platelet-derived growth factor-BB
PFA: Paraformaldehyde
PGP9.5: Protein gene product 9.5
Pref-1: Preadipocyte factor-1
RAGE: Receptor for advanced glycation endproducts
RBP4: Retinol binding protein-4
Reg3G: Regenerating islet-derived protein III-gamma
SEM: Standard error of the mean
SERCA: Sarco/endoplasmic reticulum Ca^2+^ ATPase
TIM-1: T cell immunoglobulin and mucin domain-1
TNF-α: Tumor necrosis factor-alpha
VCAM-1/CD106: Vascular cell adhesion protein-1
VEGF: Vascular endothelial growth factor
