# Supplementary material for: Sensory neuron dysfunction in orthotopic mouse models of colon cancer
Source: J Neuroinflammation. 2022 Aug 12;19:204. doi: 10.1186/s12974-022-02566-z (PMC9375288; doi:10.1186/s12974-022-02566-z)
Supplement: Supplementary file 1 — Additional file 1: Table S1. Additional table listing all differentially expressed genes in MC38 tumor-bearing versus vehicle control dorsal root ganglia. [file 12974_2022_2566_MOESM1_ESM.docx]

**Additional Table 1. Differentially expressed genes in MC38 tumor-bearing DRG**

| **Gene name** | **log_2_ fold change in MC38 versus control** | **p value** |
| --- | --- | --- |
| Acp5 | -2.8971 | 0.0063 |
| Dnah11 | -2.6631 | 0.0000 |
| Dlec1 | -2.0621 | 0.0000 |
| Col6a5 | -2.0418 | 0.0002 |
| Dnah3 | -1.7373 | 0.0004 |
| Wdr52 | -1.7241 | 0.0000 |
| Spef2 | -1.7154 | 0.0030 |
| C1qtnf3 | -1.7094 | 0.0000 |
| Ifltd1 | -1.7050 | 0.0004 |
| Fam47e | -1.6617 | 0.0005 |
| Hydin | -1.6473 | 0.0012 |
| Gm609 | -1.6152 | 0.0201 |
| Kif6 | -1.5522 | 0.0009 |
| Tsku | -1.5166 | 0.0007 |
| Ms4a1 | -1.4572 | 0.0124 |
| Igkc | -1.4367 | 0.0002 |
| Cd300lf | -1.4259 | 0.0380 |
| Wwc1 | -1.4251 | 0.0021 |
| Gm16048 | -1.4112 | 0.0060 |
| Blnk | -1.3932 | 0.0049 |
| Nespas | -1.3920 | 0.0126 |
| Rsph4a | -1.3914 | 0.0039 |
| Gpr98 | -1.3906 | 0.0043 |
| Ddo | -1.3864 | 0.0041 |
| Fbxl13 | -1.3799 | 0.0027 |
| Tmem232 | -1.3743 | 0.0033 |
| Slc7a11 | -1.3680 | 0.0005 |
| Plin4 | -1.3641 | 0.0042 |
| Dnah10 | -1.3569 | 0.0009 |
| Gm14199 | -1.3317 | 0.0137 |
| Zic5 | -1.3019 | 0.0004 |
| 1700084C01Rik | -1.2985 | 0.0016 |
| Erbb4 | -1.2750 | 0.0077 |
| 1700026D08Rik | -1.2718 | 0.0162 |
| Ccdc33 | -1.2666 | 0.0067 |
| Cyp2e1 | -1.2638 | 0.0127 |
| Tlr1 | -1.2587 | 0.0381 |
| Slc30a10 | -1.2526 | 0.0086 |
| Cd180 | -1.2342 | 0.0378 |
| Plekhn1 | -1.2332 | 0.0084 |
| **Gene name** | **log_2_ fold change** | **p value** |
| Zfp808 | -1.2238 | 0.0019 |
| Prr11 | -1.2181 | 0.0193 |
| Ctgf | -1.2142 | 0.0001 |
| Dnah6 | -1.2128 | 0.0030 |
| Gm26945 | -1.1837 | 0.0108 |
| Tnni1 | -1.1824 | 0.0059 |
| Hcar1 | -1.1809 | 0.0155 |
| Lrrc6 | -1.1799 | 0.0116 |
| Chrna3 | -1.1793 | 0.0045 |
| Rsph10b | -1.1756 | 0.0208 |
| Ttc21a | -1.1754 | 0.0255 |
| Tnc | -1.1679 | 0.0462 |
| Efcab6 | -1.1658 | 0.0112 |
| T2 | -1.1559 | 0.0348 |
| Pifo | -1.1550 | 0.0298 |
| 1700009P17Rik | -1.1543 | 0.0031 |
| Zc2hc1c | -1.1438 | 0.0030 |
| Tnfaip2 | -1.1420 | 0.0086 |
| Bcl6b | -1.1352 | 0.0132 |
| 4930485B16Rik | -1.1325 | 0.0144 |
| Il1f9 | -1.1316 | 0.0451 |
| Timp4 | -1.1291 | 0.0115 |
| Itih2 | -1.1257 | 0.0005 |
| Catip | -1.1244 | 0.0449 |
| Xlr3b | -1.1222 | 0.0000 |
| Dnaaf3 | -1.1203 | 0.0040 |
| Cfd | -1.1163 | 0.0184 |
| Fhad1 | -1.1140 | 0.0196 |
| Dpt | -1.1104 | 0.0064 |
| Sema3a | -1.1038 | 0.0105 |
| Gm15851 | -1.0964 | 0.0187 |
| Tnfsf13b | -1.0959 | 0.0130 |
| Hck | -1.0911 | 0.0320 |
| Eya1 | -1.0890 | 0.0198 |
| Tmem71 | -1.0723 | 0.0485 |
| Stk36 | -1.0598 | 0.0366 |
| Mro | -1.0590 | 0.0013 |
| Car3 | -1.0567 | 0.0046 |
| Omd | -1.0531 | 0.0118 |
| Uts2b | -1.0509 | 0.0117 |
| Pax9 | -1.0488 | 0.0157 |
| Nfkbiz | -1.0370 | 0.0284 |
| Arhgef33 | -1.0329 | 0.0429 |
| **Gene name** | **log_2_ fold change** | **p value** |
| Slc35g1 | -1.0253 | 0.0029 |
| Zic4 | -1.0182 | 0.0039 |
| Casc1 | -1.0149 | 0.0374 |
| 4930578M01Rik | -1.0121 | 0.0399 |
| Gm2366 | -1.0106 | 0.0382 |
| Slc24a4 | -1.0104 | 0.0460 |
| Gm4804 | -1.0085 | 0.0033 |
| Mpzl2 | -1.0067 | 0.0018 |
| Zc3h12a | -1.0010 | 0.0343 |
| Gm8898 | -0.9858 | 0.0139 |
| Gabrg3 | -0.9827 | 0.0244 |
| Coch | -0.9807 | 0.0097 |
| 4930533K18Rik | -0.9797 | 0.0014 |
| Ppp1r3c | -0.9667 | 0.0001 |
| Mmrn1 | -0.9657 | 0.0149 |
| Pi15 | -0.9630 | 0.0097 |
| L3hypdh | -0.9623 | 0.0439 |
| Cdr1 | -0.9597 | 0.0009 |
| Ranbp3l | -0.9543 | 0.0108 |
| Dnah2 | -0.9540 | 0.0143 |
| Wdr66 | -0.9515 | 0.0005 |
| Adrb2 | -0.9512 | 0.0075 |
| Gm15706 | -0.9508 | 0.0335 |
| Wdr96 | -0.9478 | 0.0110 |
| Tmem238 | -0.9437 | 0.0258 |
| Dnah9 | -0.9343 | 0.0256 |
| Npnt | -0.9328 | 0.0454 |
| Ikbke | -0.9257 | 0.0327 |
| Aldh1a2 | -0.9182 | 0.0115 |
| Tram2 | -0.9160 | 0.0114 |
| Cxadr | -0.9123 | 0.0056 |
| Gm27232 | -0.9107 | 0.0070 |
| Lrguk | -0.9102 | 0.0046 |
| Gm15270 | -0.9100 | 0.0324 |
| 44621 | -0.9072 | 0.0196 |
| Ror1 | -0.9059 | 0.0337 |
| Rspo3 | -0.9014 | 0.0454 |
| St8sia6 | -0.9003 | 0.0123 |
| Fap | -0.8981 | 0.0241 |
| Stra6 | -0.8979 | 0.0057 |
| Klhdc7a | -0.8927 | 0.0453 |
| Ccdc152 | -0.8906 | 0.0350 |
| Riiad1 | -0.8753 | 0.0279 |
| **Gene name** | **log_2_ fold change** | **p value** |
| Cytip | -0.8740 | 0.0484 |
| Gm16617 | -0.8729 | 0.0194 |
| Gm26773 | -0.8706 | 0.0178 |
| RP23-182N12.2 | -0.8643 | 0.0280 |
| Gjb2 | -0.8568 | 0.0477 |
| Tnfrsf11b | -0.8543 | 0.0106 |
| Aoc3 | -0.8530 | 0.0343 |
| Ighm | -0.8500 | 0.0018 |
| Zfp960 | -0.8498 | 0.0132 |
| Tc2n | -0.8483 | 0.0352 |
| Scube3 | -0.8469 | 0.0303 |
| Gm26617 | -0.8408 | 0.0402 |
| Tnfrsf13b | -0.8384 | 0.0309 |
| BC030307 | -0.8363 | 0.0309 |
| Gm16549 | -0.8335 | 0.0479 |
| Chek1 | -0.8332 | 0.0164 |
| Gm10226 | -0.8331 | 0.0315 |
| Zic1 | -0.8306 | 0.0028 |
| Gm21967 | -0.8299 | 0.0067 |
| Zic2 | -0.8200 | 0.0302 |
| Gm26778 | -0.8193 | 0.0249 |
| Gm14399 | -0.8157 | 0.0152 |
| Efcab1 | -0.8155 | 0.0128 |
| Ccdc3 | -0.8109 | 0.0197 |
| Gm17120 | -0.8042 | 0.0278 |
| BC051142 | -0.7989 | 0.0443 |
| Adh7 | -0.7953 | 0.0431 |
| Pamr1 | -0.7933 | 0.0270 |
| Sh3rf3 | -0.7932 | 0.0342 |
| Mpc1-ps | -0.7921 | 0.0010 |
| Dleu2 | -0.7872 | 0.0086 |
| Gjb6 | -0.7872 | 0.0156 |
| Efemp1 | -0.7872 | 0.0029 |
| Xrcc3 | -0.7820 | 0.0459 |
| Tmem119 | -0.7774 | 0.0348 |
| Fgfbp1 | -0.7749 | 0.0150 |
| Kcnk2 | -0.7742 | 0.0010 |
| Gm12868 | -0.7709 | 0.0427 |
| N4bp2 | -0.7692 | 0.0187 |
| Pde5a | -0.7663 | 0.0043 |
| Lbp | -0.7648 | 0.0049 |
| Ccdc170 | -0.7627 | 0.0255 |
| Igf2 | -0.7621 | 0.0331 |
| **Gene name** | **log_2_ fold change** | **p value** |
| Dcdc2a | -0.7619 | 0.0138 |
| Kctd12 | -0.7600 | 0.0227 |
| BB031773 | -0.7536 | 0.0318 |
| Slc39a8 | -0.7495 | 0.0031 |
| Fam84b | -0.7460 | 0.0285 |
| Zfp964 | -0.7456 | 0.0490 |
| Aebp1 | -0.7366 | 0.0245 |
| Filip1l | -0.7346 | 0.0203 |
| 1500015O10Rik | -0.7249 | 0.0282 |
| Ramp1 | -0.7229 | 0.0155 |
| Egln3 | -0.7223 | 0.0291 |
| Sema3d | -0.7206 | 0.0066 |
| Slc26a2 | -0.7164 | 0.0065 |
| Irak3 | -0.7157 | 0.0207 |
| Wdr89 | -0.7156 | 0.0320 |
| Fhod1 | -0.7121 | 0.0294 |
| Nphp3 | -0.7073 | 0.0052 |
| Inmt | -0.7050 | 0.0113 |
| Lrrc48 | -0.7029 | 0.0205 |
| Arhgef5 | -0.7024 | 0.0364 |
| A830080D01Rik | -0.6999 | 0.0017 |
| H19 | -0.6992 | 0.0334 |
| Cbfa2t3 | -0.6990 | 0.0490 |
| Gm12696 | -0.6985 | 0.0056 |
| Bend5 | -0.6938 | 0.0240 |
| Carf | -0.6917 | 0.0020 |
| Kif9 | -0.6889 | 0.0080 |
| Hes1 | -0.6874 | 0.0182 |
| C3 | -0.6852 | 0.0263 |
| Snx33 | -0.6822 | 0.0433 |
| Ccdc114 | -0.6790 | 0.0360 |
| Slc47a1 | -0.6771 | 0.0026 |
| Htra3 | -0.6711 | 0.0173 |
| Fn1 | -0.6673 | 0.0090 |
| Gm5069 | -0.6655 | 0.0208 |
| Hlf | -0.6652 | 0.0410 |
| Smoc2 | -0.6635 | 0.0062 |
| C030017K20Rik | -0.6605 | 0.0071 |
| A330076H08Rik | -0.6593 | 0.0248 |
| Scarf2 | -0.6567 | 0.0324 |
| Foxc1 | -0.6557 | 0.0188 |
| Ccdc80 | -0.6555 | 0.0064 |
| Pde1a | -0.6553 | 0.0285 |
| **Gene name** | **log_2_ fold change** | **p value** |
| Rarb | -0.6523 | 0.0171 |
| 9830147E19Rik | -0.6515 | 0.0328 |
| Mcc | -0.6485 | 0.0071 |
| Slc25a18 | -0.6450 | 0.0436 |
| Slco2a1 | -0.6413 | 0.0262 |
| Col3a1 | -0.6392 | 0.0099 |
| Dio2 | -0.6386 | 0.0024 |
| Clvs2 | -0.6367 | 0.0177 |
| Pltp | -0.6366 | 0.0322 |
| Ddr2 | -0.6350 | 0.0067 |
| Lca5l | -0.6340 | 0.0477 |
| Tmem39a | -0.6313 | 0.0136 |
| Hykk | -0.6303 | 0.0224 |
| RP24-74F2.12 | -0.6268 | 0.0200 |
| Timm21 | -0.6255 | 0.0147 |
| Gm13152 | -0.6233 | 0.0174 |
| Immp1l | -0.6232 | 0.0301 |
| Bgn | -0.6220 | 0.0120 |
| Lrrc17 | -0.6185 | 0.0453 |
| Gm13157 | -0.6179 | 0.0376 |
| Ecm2 | -0.6166 | 0.0444 |
| C1qtnf7 | -0.6151 | 0.0294 |
| Lekr1 | -0.6136 | 0.0437 |
| Cartpt | -0.6113 | 0.0462 |
| Cdon | -0.6085 | 0.0053 |
| mt-Nd6 | -0.6037 | 0.0271 |
| Foxp2 | -0.6026 | 0.0444 |
| Slc2a12 | -0.6020 | 0.0441 |
| Adam12 | -0.5995 | 0.0493 |
| Esr1 | -0.5952 | 0.0416 |
| Igf2bp3 | -0.5927 | 0.0406 |
| Gm26669 | -0.5919 | 0.0329 |
| Mrc2 | -0.5909 | 0.0252 |
| Adamts2 | -0.5898 | 0.0277 |
| Zcchc10 | -0.5872 | 0.0253 |
| Cdc14a | -0.5836 | 0.0414 |
| Adamts20 | -0.5826 | 0.0326 |
| Kcnk5 | -0.5825 | 0.0352 |
| Gpc6 | -0.5814 | 0.0172 |
| Crispld1 | -0.5810 | 0.0274 |
| Rbp1 | -0.5786 | 0.0397 |
| RP23-209N14.2 | -0.5779 | 0.0155 |
| Negr1 | -0.5762 | 0.0258 |
| **Gene name** | **log_2_ fold change** | **p value** |
| Sdpr | -0.5701 | 0.0338 |
| Islr | -0.5693 | 0.0214 |
| Zfp36l1 | -0.5684 | 0.0061 |
| Col12a1 | -0.5656 | 0.0099 |
| Slc2a13 | -0.5642 | 0.0429 |
| mt-Nd2 | -0.5620 | 0.0399 |
| Rab3b | -0.5599 | 0.0373 |
| Col6a1 | -0.5578 | 0.0282 |
| Lpl | -0.5554 | 0.0499 |
| Fmo2 | -0.5553 | 0.0425 |
| Zfp52 | -0.5546 | 0.0391 |
| Gm26964 | -0.5481 | 0.0368 |
| Diap3 | -0.5457 | 0.0254 |
| 9330132A10Rik | -0.5445 | 0.0091 |
| Neto2 | -0.5430 | 0.0141 |
| Ankrd44 | -0.5427 | 0.0091 |
| Zfp182 | -0.5378 | 0.0286 |
| Vcan | -0.5358 | 0.0150 |
| Cdc25b | -0.5350 | 0.0437 |
| Fat4 | -0.5331 | 0.0426 |
| Abi3bp | -0.5248 | 0.0279 |
| Zfp101 | -0.5243 | 0.0487 |
| Rdh10 | -0.5218 | 0.0342 |
| Lcor | -0.5185 | 0.0387 |
| Cacnb2 | -0.5164 | 0.0406 |
| Col1a2 | -0.5088 | 0.0499 |
| Zdhhc20 | -0.4898 | 0.0419 |
| Gm11837 | -0.4868 | 0.0198 |
| Cdk2ap2 | -0.4799 | 0.0441 |
| Pygo1 | -0.4771 | 0.0441 |
| Pitpnb | -0.4768 | 0.0059 |
| Slc38a6 | -0.4767 | 0.0424 |
| Rnf217 | -0.4759 | 0.0262 |
| Ptprb | -0.4744 | 0.0262 |
| Zfp639 | -0.4733 | 0.0462 |
| Dixdc1 | -0.4708 | 0.0337 |
| Pias2 | -0.4671 | 0.0296 |
| Wnk3 | -0.4662 | 0.0281 |
| Dnajb9 | -0.4652 | 0.0296 |
| AK129341 | -0.4619 | 0.0378 |
| Zranb3 | -0.4540 | 0.0379 |
| Sirt1 | -0.4485 | 0.0455 |
| Swt1 | -0.4484 | 0.0479 |
| **Gene name** | **log_2_ fold change** | **p value** |
| Rab4a | -0.4480 | 0.0372 |
| Fam49a | -0.4429 | 0.0491 |
| Ncam2 | -0.4415 | 0.0253 |
| Acvr2a | -0.4370 | 0.0125 |
| Nhsl1 | -0.4366 | 0.0438 |
| Tug1 | -0.4346 | 0.0160 |
| Zdhhc4 | -0.4331 | 0.0420 |
| Fbxo33 | -0.4303 | 0.0346 |
| Ptp4a2 | -0.4299 | 0.0267 |
| Ccdc88a | -0.4283 | 0.0231 |
| Ar | -0.4282 | 0.0457 |
| Nrsn1 | -0.4243 | 0.0162 |
| Zfp2 | -0.4186 | 0.0337 |
| Phf10 | -0.4159 | 0.0290 |
| Zcchc11 | -0.4156 | 0.0454 |
| Nkap | -0.4136 | 0.0481 |
| Cadm2 | -0.4136 | 0.0379 |
| Gm26809 | -0.4004 | 0.0452 |
| Tmem47 | -0.3966 | 0.0352 |
| Rsbn1 | -0.3857 | 0.0450 |
| Smndc1 | -0.3840 | 0.0481 |
| Bmpr2 | -0.3668 | 0.0212 |
| Lnp | -0.3608 | 0.0432 |
| Gm26723 | -0.3528 | 0.0400 |
| Lin7a | -0.3447 | 0.0471 |
| Cd2ap | -0.3411 | 0.0485 |
| Cdc37 | 0.3414 | 0.0446 |
| Eif3g | 0.3651 | 0.0495 |
| Pes1 | 0.3657 | 0.0402 |
| Tspan14 | 0.3738 | 0.0414 |
| Comt | 0.3741 | 0.0449 |
| H2-D1 | 0.3855 | 0.0221 |
| Rnf5 | 0.3892 | 0.0368 |
| Nkiras2 | 0.3919 | 0.0419 |
| Sra1 | 0.3929 | 0.0329 |
| Ppm1j | 0.3958 | 0.0451 |
| Fus | 0.3966 | 0.0381 |
| Fxyd2 | 0.3997 | 0.0295 |
| Sdha | 0.3999 | 0.0444 |
| Fubp3 | 0.4001 | 0.0314 |
| Mical1 | 0.4053 | 0.0343 |
| Cd82 | 0.4066 | 0.0498 |
| S100a6 | 0.4068 | 0.0369 |
| **Gene name** | **log_2_ fold change** | **p value** |
| Samm50 | 0.4086 | 0.0368 |
| Pmp22 | 0.4102 | 0.0332 |
| Kri1 | 0.4167 | 0.0410 |
| Mrps5 | 0.4184 | 0.0368 |
| Pmvk | 0.4199 | 0.0404 |
| Taf15 | 0.4218 | 0.0407 |
| Igsf11 | 0.4257 | 0.0410 |
| Plxnb3 | 0.4273 | 0.0338 |
| Ak2 | 0.4327 | 0.0363 |
| Eif6 | 0.4329 | 0.0248 |
| Ormdl3 | 0.4363 | 0.0465 |
| Smpd2 | 0.4382 | 0.0220 |
| Atox1 | 0.4383 | 0.0405 |
| Atp6v1f | 0.4411 | 0.0416 |
| Timeless | 0.4423 | 0.0419 |
| 0610039K10Rik | 0.4477 | 0.0188 |
| Mul1 | 0.4504 | 0.0347 |
| Mrps12 | 0.4518 | 0.0472 |
| Gm26566 | 0.4549 | 0.0490 |
| Cndp2 | 0.4583 | 0.0358 |
| Esam | 0.4596 | 0.0490 |
| Atp5d | 0.4644 | 0.0104 |
| Irf9 | 0.4662 | 0.0495 |
| Gm17509 | 0.4704 | 0.0158 |
| Pddc1 | 0.4739 | 0.0331 |
| Bhlhe41 | 0.4765 | 0.0384 |
| Ndufv1 | 0.4770 | 0.0070 |
| Aatf | 0.4780 | 0.0472 |
| Stx1a | 0.4786 | 0.0387 |
| Ercc1 | 0.4830 | 0.0322 |
| Anxa2 | 0.4876 | 0.0260 |
| Ing4 | 0.4892 | 0.0110 |
| Emp3 | 0.4915 | 0.0289 |
| Igtp | 0.4946 | 0.0195 |
| Il4ra | 0.4953 | 0.0456 |
| Actr8 | 0.4982 | 0.0113 |
| Plau | 0.4989 | 0.0442 |
| Adc | 0.5012 | 0.0277 |
| Smc1a | 0.5017 | 0.0428 |
| Polr3d | 0.5040 | 0.0355 |
| BC037032 | 0.5068 | 0.0223 |
| Hnrnpa0 | 0.5120 | 0.0279 |
| Cend1 | 0.5121 | 0.0097 |
| **Gene name** | **log_2_ fold change** | **p value** |
| Sssca1 | 0.5125 | 0.0198 |
| Psmc1 | 0.5157 | 0.0157 |
| Rhov | 0.5168 | 0.0352 |
| Gm10244 | 0.5210 | 0.0296 |
| Snrnp70 | 0.5223 | 0.0265 |
| 6430571L13Rik | 0.5231 | 0.0491 |
| Hip1r | 0.5278 | 0.0367 |
| C4b | 0.5280 | 0.0091 |
| Gm10543 | 0.5284 | 0.0182 |
| Nolc1 | 0.5293 | 0.0484 |
| Rlbp1 | 0.5338 | 0.0426 |
| Ndufb8 | 0.5347 | 0.0169 |
| Ttyh2 | 0.5360 | 0.0485 |
| Ebp | 0.5385 | 0.0105 |
| Stk32a | 0.5407 | 0.0237 |
| Pink1 | 0.5425 | 0.0106 |
| Sdc1 | 0.5429 | 0.0237 |
| Pim3 | 0.5435 | 0.0133 |
| Nyap1 | 0.5445 | 0.0448 |
| Htr1a | 0.5468 | 0.0467 |
| Wdr70 | 0.5479 | 0.0437 |
| Egfl8 | 0.5491 | 0.0268 |
| Fbxl19 | 0.5494 | 0.0454 |
| Sparcl1 | 0.5534 | 0.0153 |
| Gm13889 | 0.5541 | 0.0289 |
| Sart1 | 0.5563 | 0.0436 |
| Adck1 | 0.5582 | 0.0152 |
| Map1lc3a | 0.5595 | 0.0264 |
| Cdkn2d | 0.5597 | 0.0054 |
| Edf1 | 0.5611 | 0.0278 |
| Slc36a2 | 0.5681 | 0.0110 |
| Tmem158 | 0.5723 | 0.0200 |
| Ifitm7 | 0.5759 | 0.0376 |
| Nsfl1c | 0.5766 | 0.0155 |
| Ftsj3 | 0.5783 | 0.0075 |
| Eme1 | 0.5807 | 0.0389 |
| Gm9920 | 0.5840 | 0.0451 |
| Ccdc85b | 0.5902 | 0.0380 |
| Gcat | 0.5904 | 0.0090 |
| Gm14123 | 0.5942 | 0.0156 |
| Nkain1 | 0.5944 | 0.0484 |
| Ndnf | 0.6005 | 0.0286 |
| Tubb2b | 0.6024 | 0.0090 |
| **Gene name** | **log_2_ fold change** | **p value** |
| Pfdn6 | 0.6028 | 0.0418 |
| Mrpl14 | 0.6030 | 0.0070 |
| Atp6v0c | 0.6036 | 0.0097 |
| Scube1 | 0.6133 | 0.0294 |
| Sox11 | 0.6140 | 0.0140 |
| Gm20700 | 0.6141 | 0.0076 |
| Rnaseh1 | 0.6146 | 0.0199 |
| Timm13 | 0.6169 | 0.0279 |
| Ccdc12 | 0.6189 | 0.0316 |
| 1700003F12Rik | 0.6222 | 0.0285 |
| 1700034P13Rik | 0.6255 | 0.0314 |
| Snrnp35 | 0.6260 | 0.0133 |
| 1700101I11Rik | 0.6261 | 0.0210 |
| Pgf | 0.6275 | 0.0306 |
| Nptx2 | 0.6283 | 0.0286 |
| Rrp9 | 0.6296 | 0.0154 |
| Slc10a6 | 0.6306 | 0.0193 |
| Pdlim1 | 0.6307 | 0.0287 |
| Kcnmb4os1 | 0.6319 | 0.0466 |
| Gm26558 | 0.6326 | 0.0333 |
| Camk2n2 | 0.6473 | 0.0373 |
| Entpd2 | 0.6481 | 0.0168 |
| Ddx60 | 0.6532 | 0.0499 |
| Akr7a5 | 0.6541 | 0.0253 |
| Ngfrap1 | 0.6564 | 0.0243 |
| Chgb | 0.6724 | 0.0173 |
| Cox5b | 0.6763 | 0.0033 |
| Gm9767 | 0.6788 | 0.0299 |
| 9330160F10Rik | 0.6789 | 0.0467 |
| Pappa | 0.6789 | 0.0148 |
| Bst2 | 0.6856 | 0.0064 |
| Tceal5 | 0.6888 | 0.0309 |
| Alkbh4 | 0.6923 | 0.0254 |
| Ndufv3 | 0.7025 | 0.0015 |
| Polr2l | 0.7033 | 0.0241 |
| Fam71e1 | 0.7034 | 0.0340 |
| Vasp | 0.7188 | 0.0054 |
| Gm17305 | 0.7211 | 0.0082 |
| Cldn9 | 0.7232 | 0.0464 |
| Gm26586 | 0.7280 | 0.0137 |
| Arxes2 | 0.7302 | 0.0074 |
| Ccs | 0.7303 | 0.0096 |
| Fst | 0.7363 | 0.0319 |
| **Gene name** | **log_2_ fold change** | **p value** |
| Mag | 0.7372 | 0.0328 |
| Galnt6 | 0.7445 | 0.0481 |
| Tceal6 | 0.7450 | 0.0039 |
| 5330417C22Rik | 0.7516 | 0.0407 |
| Gm26584 | 0.7603 | 0.0394 |
| AI115009 | 0.7725 | 0.0232 |
| Maff | 0.7904 | 0.0365 |
| Oasl2 | 0.7960 | 0.0174 |
| Tceal3 | 0.7999 | 0.0120 |
| Rpp25l | 0.8014 | 0.0041 |
| Rps28 | 0.8121 | 0.0449 |
| Gm9800 | 0.8131 | 0.0475 |
| Asphd1 | 0.8135 | 0.0218 |
| Gm17111 | 0.8136 | 0.0287 |
| Zmynd15 | 0.8155 | 0.0278 |
| Ndufa3 | 0.8213 | 0.0005 |
| Gm17641 | 0.8245 | 0.0018 |
| Bdnf | 0.8316 | 0.0016 |
| Cxcl10 | 0.8433 | 0.0116 |
| Hba-a2 | 0.8466 | 0.0151 |
| Wfdc2 | 0.8484 | 0.0454 |
| 1700120B22Rik | 0.8511 | 0.0314 |
| Plvap | 0.8568 | 0.0127 |
| I830012O16Rik | 0.8732 | 0.0330 |
| Gm12356 | 0.8756 | 0.0192 |
| Batf2 | 0.8769 | 0.0301 |
| Ifit1 | 0.8834 | 0.0253 |
| Avpr1a | 0.8973 | 0.0423 |
| Fam150b | 0.9014 | 0.0037 |
| Tmem258 | 0.9028 | 0.0140 |
| Them5 | 0.9030 | 0.0210 |
| Socs3 | 0.9033 | 0.0452 |
| Exoc3l2 | 0.9209 | 0.0341 |
| Rps29 | 0.9410 | 0.0091 |
| Ifi27l2a | 0.9425 | 0.0310 |
| Usp18 | 0.9429 | 0.0090 |
| Gm4419 | 0.9446 | 0.0231 |
| Oas1a | 0.9526 | 0.0401 |
| Kcnd3os | 0.9581 | 0.0208 |
| RP24-266F18.1 | 0.9624 | 0.0233 |
| Arxes1 | 0.9653 | 0.0067 |
| Steap1 | 0.9698 | 0.0329 |
| Ifit3 | 0.9754 | 0.0224 |
| **Gene name** | **log_2_ fold change** | **p value** |
| Itk | 0.9830 | 0.0046 |
| G730003C15Rik | 0.9841 | 0.0167 |
| Fgfbp3 | 0.9869 | 0.0316 |
| Chaf1b | 0.9925 | 0.0448 |
| Myrf | 1.0051 | 0.0228 |
| Mcm5 | 1.0084 | 0.0039 |
| Gm26536 | 1.0154 | 0.0477 |
| Fam222a | 1.0315 | 0.0025 |
| Dcst1 | 1.0367 | 0.0298 |
| Kcnj11 | 1.0475 | 0.0333 |
| Abhd11os | 1.0599 | 0.0093 |
| Irf7 | 1.0607 | 0.0069 |
| Dab1 | 1.0628 | 0.0333 |
| Srcrb4d | 1.0642 | 0.0145 |
| Fgg | 1.0651 | 0.0286 |
| 2610034M16Rik | 1.0806 | 0.0046 |
| Neurog3 | 1.0880 | 0.0267 |
| Gm26845 | 1.0899 | 0.0217 |
| N4bp3 | 1.0905 | 0.0352 |
| Mapk13 | 1.1183 | 0.0184 |
| Gm20319 | 1.1278 | 0.0120 |
| Fam166a | 1.1435 | 0.0001 |
| Oas2 | 1.1489 | 0.0420 |
| Vgf | 1.1577 | 0.0032 |
| Klhl33 | 1.1591 | 0.0075 |
| Acpt | 1.1663 | 0.0216 |
| 4933428G20Rik | 1.1692 | 0.0008 |
| Gm9885 | 1.1924 | 0.0019 |
| AY036118 | 1.2017 | 0.0007 |
| Fam167b | 1.2051 | 0.0246 |
| Oasl1 | 1.2280 | 0.0309 |
| Isg15 | 1.2409 | 0.0022 |
| 1700069B07Rik | 1.2678 | 0.0103 |
| Adamts8 | 1.2821 | 0.0003 |
| Esrrb | 1.3177 | 0.0134 |
| Ryr1 | 1.3289 | 0.0113 |
| RP23-446G23.1 | 1.4136 | 0.0002 |
| Gm9939 | 1.4297 | 0.0028 |
| Lrg1 | 1.4407 | 0.0005 |
| Grrp1 | 1.4493 | 0.0009 |
| Tmem173 | 1.4985 | 0.0001 |
| Mei1 | 1.6356 | 0.0013 |
