# Supplementary material for: Sensory neuron dysfunction in orthotopic mouse models of colon cancer
Source: J Neuroinflammation. 2022 Aug 12;19:204. doi: 10.1186/s12974-022-02566-z (PMC9375288; doi:10.1186/s12974-022-02566-z)
Supplement: Supplementary file 2 — Additional file 2: Tables S2 and S3. Additional tables listing all detected inflammatory mediators in cancer cell growth medium and tumor-bearing mouse serum. [file 12974_2022_2566_MOESM2_ESM.docx]

**Additional Table 2. MC38 and CT26 cell secreted factors**

| Factors released by **MC38** cells only | Factors released by both cell lines* | Factors released by **CT26** cells only | |
| --- | --- | --- | --- |
| CCL11/Eotaxin | Amphiregulin | CCL17/TARC | Pref-1/DLK-1/FA1 |
| Chitinase 3-like 1 | **CCL2/JE/MCP-1** | BAFF/BLyS/TNFSF13B | P-Selectin/CD62P |
| FGF-21 | CCL5/RANTES | CCL19/MIP-3ß | RAGE |
| Proliferin | CX3CL1/Fractalkine | Coagulation Factor III | RBP4 |
| WISP-1/CCN4 | **CXCL1/KC** | Complement Component C5 | Reg3G |
|  | **CXCL10/IP-10** | CXCL2/MIP-2 | Resistin |
|  | Cystatin C | Endostatin | Serpin F1/PEDF |
|  | Flt-3 Ligand | E-Selectin/CD62E | TIM-1/KIM-1/HAVCR |
|  | GDF-15 | IGFBP-1 |  |
|  | IGFBP-6 | IGFBP-3 |  |
|  | LDL R | IL-11 |  |
|  | M-CSF | IL-12 p40 |  |
|  | Myeloperoxidase | IL-1α/IL-1F1 |  |
|  | Osteopontin (OPN) | IL-1β/IL-1F2 |  |
|  | Proprotein Convertase 9/PCSK9 | IL-2 |  |
|  | **Serpin E1/PAI-1** | IL-27 p28 |  |
|  | VCAM-1/CD106 | LIF |  |
|  | VEGF | LIX |  |
|  |  | MMP-3 |  |
|  |  | MMP-9 |  |
|  |  | Osteoprotegerin/TNFRSF11B |  |
|  |  | Periostin/OSF-2 |  |

*Factors highlighted in **bold** were detected in tumor-bearing mouse serum.

**Additional Table 3. Factors elevated in serum of MC38 and CT26 tumor-bearing mice**

| Factors upregulated in **MC38** mouse serum | Factors upregulated in **both** sera* | Factors upregulated in **CT26** mouse serum | |
| --- | --- | --- | --- |
| Angiopoietin-2 | BAFF/BLyS/TNFSF13B | Adiponectin/Acrp30 | P-Selectin/CD62P |
| CCL6/C10 | **CCL2/JE/MCP-1** | Amphiregulin | RAGE |
| CXCL16 | CCL20/MIP-3a | Angiopoietin-1 | Thrombopoietin |
| Endoglin/CD105 | CCL3/CCL4/MIP-1a/ß | CCL11/Eotaxin | TNF-a |
| Gas 6 | **CXCL1/KC** | CCL12/MCP-5 | VEGF |
| G-CSF | **CXCL10/IP-10** | CCL19/MIP-3ß |  |
| GDF-15 | CXCL2/MIP-2 | CCL22/MDC |  |
| IGFBP-1 | EGF | CCL5/RANTES |  |
| IL-28A/B | FGF acidic | CD14 |  |
| Osteopontin (OPN) | GM-CSF | Coagulation Factor III |  |
| Pentraxin 2/SAP | HGF | CX3CL1/Fractalkine |  |
| Proliferin | IFN-γ | CXCL9/MIG |  |
| Proprotein Convertase 9/PCSK9 | IL-1α/IL-1F1 | DKK-1 |  |
|  | IL-23 | IL-10 |  |
|  | IL-6 | IL-13 |  |
|  | Lipocalin-2/NGAL | IL-17A |  |
|  | Osteoprotegerin/TNFRSF11B | IL-2 |  |
|  | PDGF-BB | IL-22 |  |
|  | **Serpin E1/PAI-1** | IL-3 |  |
|  |  | IL-5 |  |
|  |  | Leptin |  |
|  |  | LIX |  |
|  |  | MMP-9 |  |
|  |  | PD-ECGF/Thymidine phosphorylase |  |
|  |  | Pentraxin 3/TSG-14 |  |
|  |  | Pref-1/DLK-1/FA1 |  |

*Factors highlighted in **bold** were detected in cancer cell-conditioned media.
