# Supplementary figures and images for: Sensory neuron dysfunction in orthotopic mouse models of colon cancer
Source: J Neuroinflammation. 2022 Aug 12;19:204. doi: 10.1186/s12974-022-02566-z (PMC9375288; doi:10.1186/s12974-022-02566-z)

## Lumbar DRG $\text{Ca}^{2+}$ Imaging

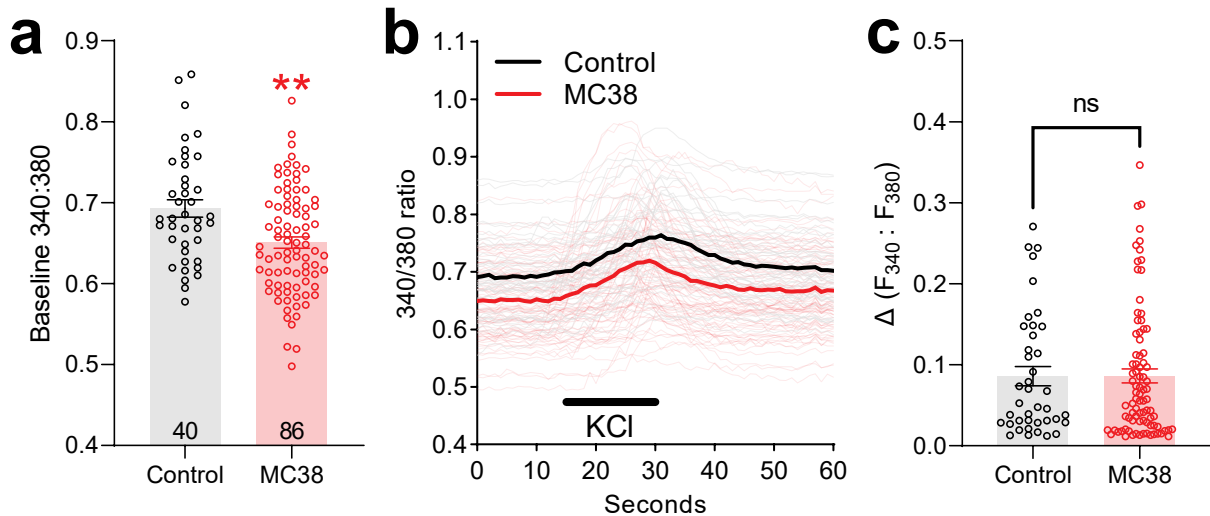

## Non-Lumbar DRG $\text{Ca}^{2+}$ Imaging

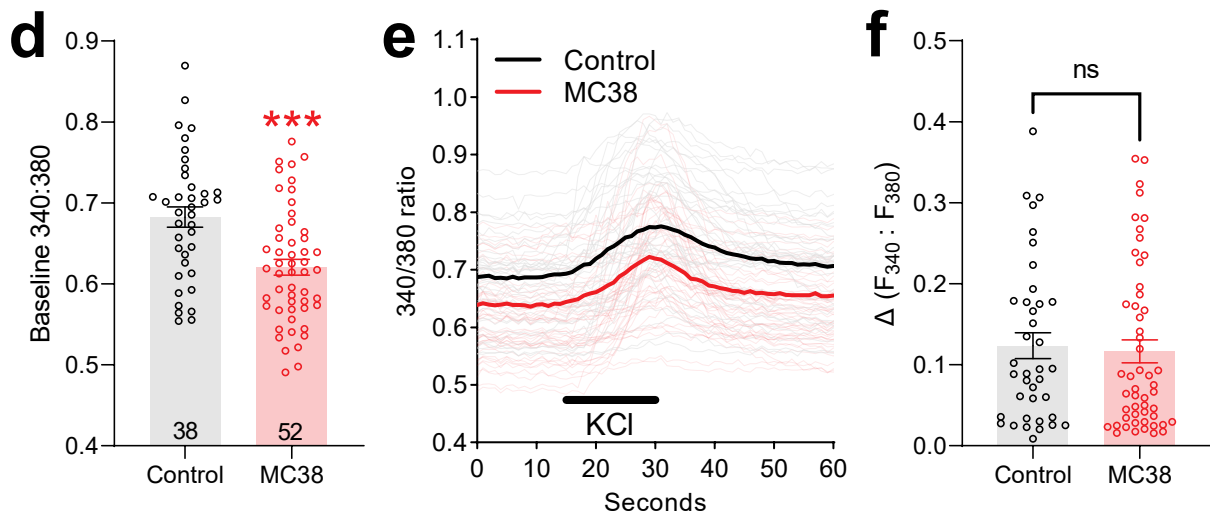

Supplement: Supplementary file 3 — Additional file 3: Figure S1. Additional figure showing the results of live-cell Ca2+ imaging, presenting the separately analyzed DRG neurons isolated from lumbar a–c and non-lumbar (d–f). A similar decrease in [Ca2+]i was observed in DRG neurons from both levels in tumor-bearing mice compared to control, 3 weeks after tumor inoculation. [file 12974_2022_2566_MOESM3_ESM.pdf]
