# Supplementary material for: Sensory neuron dysfunction in orthotopic mouse models of colon cancer
Source: J Neuroinflammation. 2022 Aug 12;19:204. doi: 10.1186/s12974-022-02566-z (PMC9375288; doi:10.1186/s12974-022-02566-z)

a

Disease and Function enrichment: MC38

Category	p-value (range)	Number of genes
Cancer	2.86E-05-4.64E-02	23
Dermatological Diseases and Conditions	2.86E-05-4.64E-02	20
Hematological Disease	2.86E-05-4.26E-02	14
Immunological Disease	2.86E-05-3.47E-02	13
Organismal Injury and Abnormalities	2.86E-05-4.73E-02	23

Disease and Function enrichment: Vehicle

Category	p-value (range)	Number of genes
Developmental Disorder	2.29E-10-5.79E-03	17
Hereditary Disorder	2.29E-10-6.32E-03	22
Organismal Injury and Abnormalities	2.29E-10-8.68E-03	67
Respiratory Disease	2.29E-10-8.68E-03	41
Cellular Function and Maintenance	6.04E-09-7.57E-03	12

b

Cancer-related genes

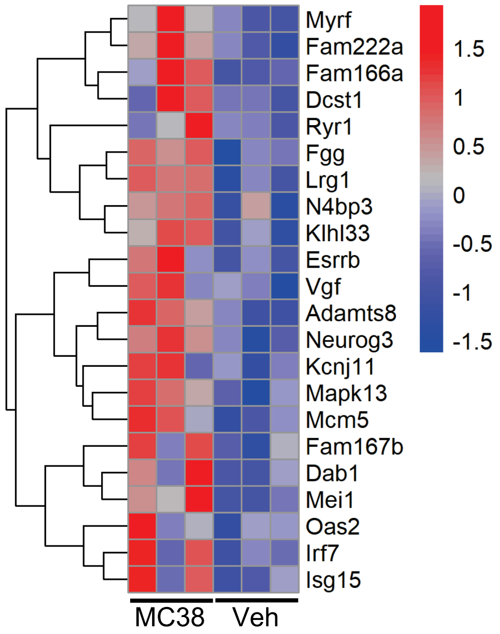

Supplement: Supplementary file 4 — Additional file 4: Figure S2. Additional figure showing disease and functional enrichment analysis of RNA sequencing of DRGs from MC38-injected mice. [file 12974_2022_2566_MOESM4_ESM.pdf]
